# Models of Intervention: Helping Agents and Human Users Avoid Undesirable Outcomes

**DOI:** 10.3389/frai.2021.723936

**Published:** 2022-02-03

**Authors:** Sachini Weerawardhana, Darrell Whitley, Mark Roberts

**Affiliations:** ^1^Department of Computer Science, Colorado State University, Fort Collins, CO, United States; ^2^The U.S. Naval Research Laboratory, Washington, DC, United States

**Keywords:** plan recognition, automated planning, behavior classification, intervention, human-aware intervention

## Abstract

When working in an unfamiliar online environment, it can be helpful to have an observer that can intervene and guide a user toward a desirable outcome while avoiding undesirable outcomes or frustration. The *Intervention Problem* is deciding when to intervene in order to help a user. The Intervention Problem is similar to, but distinct from, Plan Recognition because the observer must not only recognize the intended goals of a user but also when to intervene to help the user when necessary. We formalize a family of Intervention Problems and show that how these problems can be solved using a combination of Plan Recognition methods and classification algorithms to decide whether to intervene. For our benchmarks, the classification algorithms dominate three recent Plan Recognition approaches. We then generalize these results to Human-Aware Intervention, where the observer must decide in real time whether to intervene human users solving a cognitively engaging puzzle. Using a revised feature set more appropriate to human behavior, we produce a learned model to recognize when a human user is about to trigger an undesirable outcome. We perform a human-subject study to evaluate the Human-Aware Intervention. We find that the revised model also dominates existing Plan Recognition algorithms in predicting Human-Aware Intervention.

## 1. Introduction

Even the best plan can go wrong. Dangers arise from failing to execute a plan correctly or as a result of actions executed by a nefarious agent. Consider route planning where a driver is unaware of upcoming road damage or a traffic jam. Or consider cyber-security where a user is unaware of an unsafe hyperlink. In both, plans achieving the desirable goal have similar prefixes to those that result in undesirable outcomes. Suppose an observer watches the actions of a user working in a risky environment where plans may be subverted to reach an undesirable outcome. We study the problem of how the observer decides whether to intervene if the user appears to need help or the user is about to take an action that leads to an undesirable outcome.

We introduce *Plan Intervention* as a new computational problem and relate it to plan recognition. The Plan Intervention problem can be thought of as consisting of two sub-problems: (1) *Intervention Recognition* and (2) *Intervention Recovery*. In the Intervention Recognition phase, the observer needs to make a decision whether or not the user's likely plan will avoid the undesirable state. Thus, it is possible to argue that if the observer implements an existing state-of-the-art plan recognition algorithm (e.g., Plan Recognition as Planning Ramırez and Geffner, 2009), then intervention can take place when the likely goal of the recognized plan satisfies the undesirable state.

In fact, using existing plan recognition algorithms to identify when intervention is required has been studied in several works (Pozanco et al., 2018; Shvo and McIlraith, 2020). A key strategy in these solutions is to reduce the pending goal hypotheses set to expedite recognition. In this paper, we propose a different approach to perform recognition where the observer uses automated planning combined with machine learning to decide whether the user must be interrupted to avoid the undesirable outcome. Our long term objective is to develop this intervention model as an assistive teaching technology to gradually guide human users through cognitively engaging tasks. To this end, for the Intervention Recovery phase we want to enable the observer to actively help the human user recover from intervention and continue the task. We leave Intervention Recovery for future work.

We show that the Intervention Problem carries subtleties that the state-of-the-art Plan Recognition Algorithms do not address where it is difficult for the observer to disambiguate the desirable and the undesirable outcomes. We propose two complementary solutions for Plan Intervention: (1) Unsafe Suffix Intervention and (2) Human-aware Intervention. We show that these two complementary solutions dominate state-of-the-art plan recognition algorithms in correctly recognizing when intervention is required.

Typically, the state-of-the-art plan recognition algorithms reconstruct plan hypotheses from observations. Ramırez and Geffner (2009) and Ramırez and Geffner (2010) reconstruct plan hypotheses by *compiling the observations away* and then using an automated planner to find plans that are compatible with the observations. Another approach generates the plan hypotheses by concatenating the observations with a projected plan obtained from an automated planner (Vered and Kaminka, 2017). The costs of the reconstructed plan hypotheses then lead to a probability distribution over likely goals of the user. Existing plan recognition algorithms require that prior probabilities of likely goals be provided as input.

For intervention, providing goal priors is difficult because certain facts about the domain are hidden to the user and unintended goals may be enabled during execution regardless of the priors. Furthermore, human actors may not construct plans the same way as an automated planner. They may make mistakes early on during tasks having a steep learning curve. Partial knowledge about the domain may preclude the human user from knowing the full effects of his actions or he may not be thinking about the effects at all. Therefore, the observer may not always be able to accurately estimate what the users are trying to do.

We study two kinds of Intervention Problems. In *Unsafe Suffix Intervention*, the observer uses automated planners to project the remaining suffixes and extract features that can differentiate between safe and unsafe plans. We evaluate the recognition accuracy of Unsafe Suffix Intervention on benchmark planning problems. In *Human-aware Intervention*, the observer uses the observed partial solution to extract features that can separate safe and unsafe solutions. We evaluate the accuracy of Human-aware Intervention on a new Intervention Planning benchmark called Rush Hour.

The contributions of this paper are:

- formalizing the online Intervention Problem to determine when to intervene;
- modeling the observer's decision space as an Intervention Graph;
- defining features to assess the criticality of a state using the Intervention Graph and the sampled plans;
- extending existing benchmarks by Ramırez and Geffner (2009; 2010) to incorporate Intervention and evaluating our intervention approach for the extended benchmarks;
- introducing a new Plan Intervention benchmark domain called *Rush Hour*. This is a cognitively engaging puzzle solving task where a player moves vehicles arranged on a grid to clear a path for a target vehicle.
- formalizing the Human-aware Intervention Problem for the Rush Hour planning task and designing features to estimate the criticality using behavior features derived from the observed partial plan;
- presenting the results from a human-subjects study where we collected human behavior on Rush Hour;
- extending an existing plan recognition model to the Intervention Problem; and
- training and evaluating the classification models using Rush Hour puzzle solutions collected from a human subject experiment and showing that the approach works well for the Rush Hour problem.

The rest of this paper is organized as follows. In Section 2, we distinguish intervention from plan recognition. In Section 3 we define a general form of the Intervention Problem and introduce three variants: (1) Intervention for a Single User, (2) Intervention in the Presence of a Competitor and (3) Human-aware Intervention. In Section 4, we present approaches for Intervention for a Single User and Intervention in the Presence of a Competitor, both of which use the Intervention Graph and the sampled plans to recognize unsafe suffixes. Section 5 presents our evaluation of Unsafe Suffix Intervention. We compare the accuracy of our proposed algorithms against the state-of-the-art plan recognition algorithms on planning domains from the International Planning Competition (IPC). In Section 6, we present Human-aware Intervention, that uses machine learning to determine when to intervene. We introduce a new planning domain called Rush Hour and study how human users solve Rush Hour puzzle as a planning task. We discuss the accuracy of Human-aware Intervention compared to the state-of-the-art plan recognition algorithms when predicting intervention for human users in Section 7. In Section 8, we discuss the state-of-the-art in plan and goal recognition and human user behavior classification. The discussion in Section 9 presents our analysis on why plan recognition falls short in solving Intervention Problems. Section 10 presents the open questions for future research in designing human-aware Intervention models. Section 11 details the human subject experiment we conducted to collect the data required for Human-aware Intervention.

## 2. Distinguishing Intervention From Plan Recognition

We model **intervention** in environments where a *user* is trying to achieve desirable goal(s), denoted d, while avoiding undesirable outcomes, denoted u. Some environments include a *competitor* who may also take actions in the world, but we assume the user is not aware of the competitor's actions.

We define the *observer* to be the intervening assistant agent. An *observer* receives each action and decides whether to 1) intervene, or 2) to allow the action to be executed. The observer holds a history of previous observations *H* = (*o*_1_, *o*_2_, …, *o*_*i*−1_) that indicate the actions executed by the user or competitor. Based on these observations, the observer must decide the user is about to do something unsafe (u) or is moving too far away from a desirable goal (d) by creating a projection of possible actions. We call such a projection a *suffix*. We denote a single *suffix* projection as *X* and the set of projections as $X_{◇}$ because there will usually be many projections.

At first glance, it might seem that intervention is a variant of Plan Recognition for d and u. However, there are several subtleties that make intervention unique, which we now discuss.

- **Intervention is an online problem**. In most cases Plan Recognition (e.g., Ramırez and Geffner, 2009, 2010; Sohrabi et al., 2016a) is an offline problem; there are a few notable exceptions (Mirsky et al., 2018). However, intervention is inherently online and dynamic. The observer decides whether to intervene (or not) every time the user(s) presents an action *o*_*i*_. In order to make the decision, the observer uses the observation history *H*, which contains accepted actions. Intervention is a multi-agent problem as well. This has ramifications in environments where the user and the competitor compete to achieve close but different goals. With intervention, the observer can help the user by accepting actions into *H* that only help further the user's goal. This is not possible with offline Plan recognition.
- **Agents may have distinct views of the problem**. The user and the competitor are modeled with different domain definitions. The user wants to satisfy the desirable state (d), while the observer wants to avoid the *hidden* undesirable state (u). The competitor is trying to subvert the user's goal by enabling preconditions for u without the user's knowledge. This follows from many real world applications such as cyber-security, where an attacker sends an email to trick the user into visiting a phishing website and reveal a password. When the user and the competitor (if present) reveal their plan(s) incrementally, the observer needs to decide whether the revealed actions make it impossible for the user to avoid state u considering the plans from the user's and the competitor's domain definitions collectively. Any action that make it impossible for the user to avoid u must be for flagged for intervention.
  We cannot assume that only the competitor's actions will satisfy u. In the cyber-security example, the attacker only sends the click-bait email. The user, while executing routine tasks on the computer, in fact follows the link and submits password to the phishing web site satisfying u. If the plans for u and d share a long common prefix, it may be difficult for the observer to disambiguate between the goals in time to help the user avoid u.
- **Partitioned suffixes**. The observer should allow the user to pursue suffixes leading to d and intervene when actions are presented from suffixes that get “too close” to u. Our key insight is to model the “goals” of the user and competitor, which justifies our use of planning to find these suffixes. The observer needs to consider two kinds of goals that might require intervention:
  1. Cases where the user is headed toward an undesirable outcome u. These cases can be solved by plan recognition.
  2. Cases where the user unwittingly enables an undesirable outcome u by taking actions toward a desired outcome d. There is an inherent trade-off between intervening and allowing the user some freedom to pursue d. Suppose that some suffix *X*_u_ leads to u and suffix *X*_d_ leads to d. Then it can happen that by simply following the plan leading to d, the user enables u when there is enough overlap between *X*_u_ and *X*_d_. To manage these cases, we must consider plans leading to both d and u together. We use the notation u ∪ d to identify when the satisfying d also satisfies u as a side effect. A planning problem with u ∪ d as the goal must generate plans that satisfy both u and d as solutions. The recognition approach we present in this paper focuses on identifying plan suffixes that satisfy d and also will satisfy u as a side effect. Our approach relies on features extracted from the planning problem representation to generate learned models that can solve this recognition problem.
  Partitioning the suffixes allows the observer to learn the differences between the safe and the unsafe suffixes and balance specific unsafe actions against allowing users to pursue their goals. For example, in a malicious email attack such as phishing, the user will still want to check email. When u and d are too close, disambiguation based on plan cost may not be sufficient (we will demonstrate with an example shortly).
- **Goal priors cannot be estimated reliably**. Plan Recognition algorithms use a prior probability distribution over the goal hypotheses to estimate the posterior probability of the likely goals given the observations. We assume the user does not intend to reach the undesired state u (i.e., prior probability ≈ 0). In contrast, a competitor does intend to achieve u (i.e., prior probability ≈ 1). In general, if the priors are not accurate, it can be difficult to disambiguate between plans that reach desirable and undesirable goal states.
- **Emphasis on suffix analysis**. In Plan Recognition, the observer uses *H* to derive the user's likely plan. *H* can be either an ordered sequence of actions (Ramırez and Geffner, 2009, 2010) or an ordered sequence of states (Sohrabi et al., 2016a). Here, our approach deviates from existing plan recognition problem formulation. In the first form of intervention, the observer considers the remaining plans (i.e., suffixes) in safe and unsafe partitions (instead of *H*) to learn to recognize unsafe suffixes in order to help the user avoid u. We call the first intervention model, *Unsafe Suffix Intervention*. In the second form of intervention, the observer learns to recognize that the user is not making progress toward d by analyzing the *H*. However, in contrast to existing plan recognition, which uses the plan cost to recognize the user's plan, we use domain-specific features to recognize in advance that the user's current plan will satisfy u. In the next phase of this work, we intend to study how to enable the observer to help the user recover from intervention to *guide* the user toward d. The second intervention model is particularly useful when the user is a human, who may not precisely follow an optimal plan. Therefore, we call the second intervention model, *Human-aware Intervention*.

**Figure 1:**
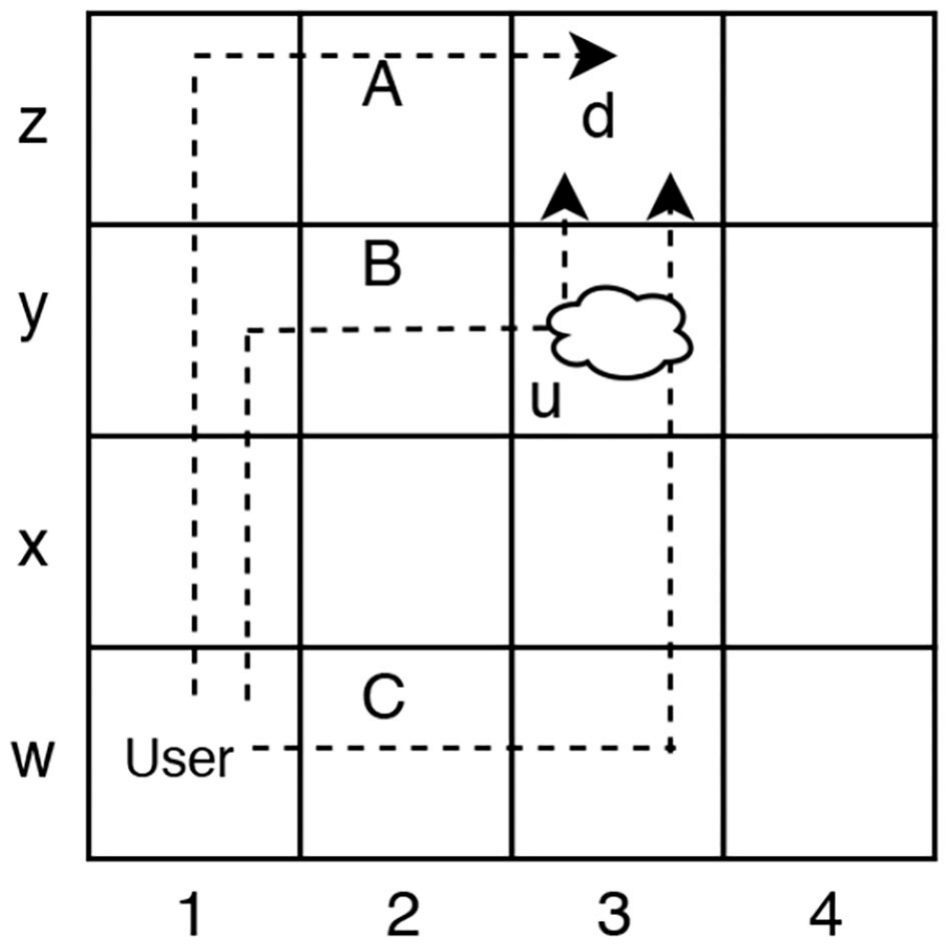
In this Grid Navigation domain, a rational user who is unaware of u, may choose to execute plans A, B, or C because they are of equal (optimal) cost. Observer must recognize that intervention is required if the user is executing plans B or C before u is satisfied.

We will present two examples for *Unsafe Suffix Intervention*. The Grid Navigation domain example is used to illustrate intervention with the user and observer. In the grid navigation task illustrated in Figure 1, the user navigates from W1 to Z3 by moving vertically and horizontally and Y3 contains a pit the user cannot see: d= (AT Z3) and u= (AT Y3). Plans corresponding to paths A, B, C are all feasible solutions to the user's planning task. However, plans B and C are unsafe because they satisfy {u ∪ d}.

Table 1 shows how an observer modeled as an offline Plan Recognition agent will recognize the goals given observations *O* for the Grid Navigation problem in Figure 1. Let us assume that the observer implements the Plan Recognition as Planning algorithm introduced by Ramırez and Geffner (2010) to disambiguate between u and d. They show that the most likely goal will be the one that minimizes the cost difference $c\left(\text{g}|O\right)-c\left(\text{g}|\bar{O}\right),\text{g}\in \left\{\text{d},\text{u}\right\}$. For each incrementally revealed *O* (shown in columns), the observer finds the most likely goal that agrees with *O*. We assumed that the user is following a satisficing plan to achieve goals. For the Grid Navigation example, the observer cannot correctly disambiguate between d and u. More importantly, the final last column satisfies u and it is too late for the user to avoid u.

**Table 1:** Observer modeled as a plan recognition agent for the grid navigation example in Figure 1.

**O**	**(Move w1 x1)**	**(Move w1 x1** **Move x1 y1)**	**(Move w1 x1** **Move x1 y1** **Move y1 y2)**	**(Move w1 x1** **Move x1 y1** **Move y1 y2** **Move y2 y3)**
$c(\text{u}|O)-c(\text{u}|\bar{O})$	4−4 = 0	4−4 = 0	4−4 = 0	4−4 = 0
$c(\text{u}|O)-c(\text{u}|\bar{O})$	5−5 = 0	5−5 = 0	5−5 = 0	5−5 = 0
Most likely goal	No decision	No decision	No decision	Fail

**Figure 2:**
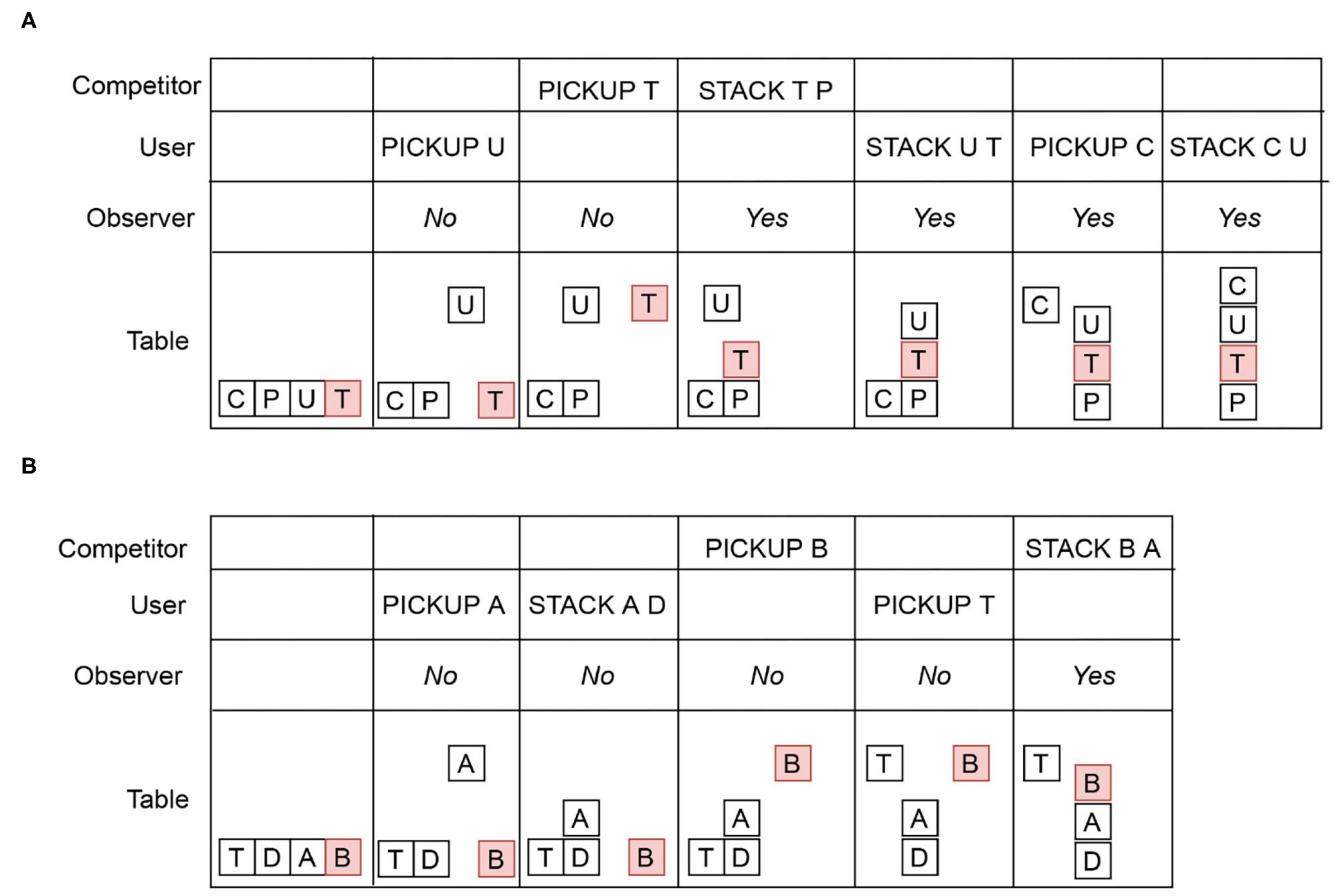
**(A)** User and competitor intervention modeled in the Blocks Words domain, where u= (CUT) and d= (CUP). **(B)** User and competitor intervention where u= (BAD) and d= (TAD). Initially, all four blocks are on the table. An action in the user or competitor row indicates the Intervention Suffix. The *Yes* label in the observer's row indicates that intervention is required. The *No* label indicates that intervention is not required.

Now let us consider a situation where there may be some additional agent. For example, in a cyber-security application, a second agent may insert malicious code in a file to gain access to a privileged information. More generally, we call this additional agent a competitor, since it is not always the case that they are “attacking” a user. The Blocks Word domain example from Ramırez and Geffner (2009) is used to illustrate intervention with the user, the competitor and the observer. In the Intervention Problem illustrated in Figure 2A, the competitor's goal u= {(CLEAR C)(ON C U)(ON U T)}(i.e., CUT) and the user's goal d = {(CLEAR C)(ON C U)(ON U P)}(i.e., CUP). The user cannot recognize the block T (shown in red). The competitor only modify the state of block T and executes actions with the block T. As shown in the first column, initially, all four blocks are on the table. Both the user and the competitor incrementally reveal their plans. The user's and the competitor's rows in Figure 2A show a *reveal sequence* from left to right. The row for the table shows the resulting states after each reveal. The observer needs to recognize that when the competitor reveals STACK T P, it becomes impossible for the user to avoid u. However, the user may continue to reveal actions because he cannot recognize the post-condition of STACK T P. The *Yes* labels in the observer's row indicate that intervention is required. Note that in the Blocks Words example, d and u are distinct enough to disambiguate early.

Table 2 shows how an observer modeled as an offline Plan Recognition agent will recognize the goals for the Blocks Words problem in Figure 2A. Similar to the Grid Navigation example, the Plan Recognition agent implements the Plan Recognition as Planning algorithm introduced by Ramırez and Geffner (2010) to disambiguate between u and d. All other assumptions in the Grid Navigation Plan Recognition task also hold in this problem. The observer distinguishes u and d somewhat better than the Grid Navigation example, because the goals are different enough. The most likely goal identified by the observer aligns with the Yes/No decisions in Figure 2A. However, in the last reveal, the observer correctly identifies u as the goal. However, the observer cannot help the user avoid u in the final reveal because *O* satisfies u by definition of *O* in Plan Recognition as Planning. With the proposed intervention algorithms, which learn the distinctions between unsafe and safe plans, we hope to improve the intervention recognition accuracy for the observer, while allowing the user some freedom to satisfy d avoiding u. Furthermore, by accepting actions that only help the user advance toward d safely into the observation history *H*, our intervention algorithm ensures that the user avoids u.

**Table 2:** Observer modeled as a Plan Recognition agent for the Blocks Words example in Figure 2A.

**O**	**(PICKUP U)**	**(PICKUP U** **PICKUP T)**	**(PICKUP U** **PICKUP T** **STACK T P)**	**(PICKUP U** ** PICKUP T** **STACK T P** **STACK U T)**	**(PICKUP U** **PICKUP T** **STACK T P** **STACK U T** ** PICKUP C)**	**(PICKUP U** **PICKUP T** **STACK T P** **STACK U T** ** PICKUP C** **STACK C U)**
$c\left(\text{u}|O\right)-c\left(\text{u}|\bar{O}\right)$	4−4 = 0	8−4 = 4	8−4 = 4	8−4 = 4	8−4 = 4	8−4 = 4
$c\left(\text{d}|O\right)-c\left(\text{d}|\bar{O}\right)$	4−4 = 0	6−4 = 2	10−4 = 6	16−4 = 12	18−4 = 14	20−4 = 16
Most likely goal	No decision	d	u	u	u	Fail

## 3. Defining Intervention Problems

In this section we outline our main assumptions (Section 3.1), discuss the STRIPS planning model (Section 3.2), discuss an important notion of direct or indirect actions leading to u (Section 3.3), and define the general form of the Intervention Problem as well as highlight the family of problems we study in this paper (Section 3.4).

### 3.1. Preliminaries and Assumptions

Because the observer's objective is to help the user safely reach d, an intervention episode (i.e., a sequence of intervention decisions) is defined from the initial state *s*_0_ until d is satisfied. If the competitor is present, the observer decides which actor's presented action to process first, randomly. The actor(s) take turns in presenting actions from their respective domain definitions to the observer until the intervention episode terminates.

We make some simplifying assumptions in this study. **Observability:** The observer has full observability, and knows about states d and u. u is unknown to the user. d is unknown to the competitor. When we say *unknown*, it means that the agent does not actively execute actions to enable the unknown goal. The user cannot recognize the effects of a competitor's actions. **Plans:** The user follows a satisficing plan to reach d, but may reach u unwittingly. There is a satisficing plan to reach u ∪ d and we assume that it has a common prefix with a plan to reach d. We assume that the user continues to present the observer with actions from his original plan even after the first positive flag and does not re-plan. **Competitor:** When present, the competitor only performs actions using objects hidden to the user; this restriction follows from many security domains where an attacker is a remote entity that sets traps and expects the user to become an unwitting accomplice. The user and the competitor are (bounded) rational agents.

### 3.2. The Intervention Model

We model the users in the intervention environment as STRIPS planning agents (Fikes and Nilsson, 1971). A STRIPS planning domain is a tuple *D* = 〈*F, A, s*_0_〉 where *F* is the set of fluents, *s*_0_ ⊆ *F* is the initial state, and *A* is the set of actions. Each action *a* ∈ *A* is a triple *a* = 〈*Pre*(*a*), *Add*(*a*), *Del*(*a*)〉 that consists of preconditions, add and delete effects, respectively, where *Pre*(*a*), *Add*(*a*), *Del*(*a*) are all subsets of *F*. An action *a* is applicable in a state *s* (represented by subsets of *F*) if preconditions of *a* are true in *s*; *pre*(*a*) ∈ *s*. If an action *a* is executed in state *s*, it results in a new state *s*′ = (*s*\*del*(*a*) ∪ *add*(*a*)), and defined by the state transition function γ(*s, a*) = *s*′. A STRIPS planning problem is a tuple *P* = 〈*D, G*〉, where *D* is the STRIPS planning domain and *G* ⊆ *F* represents the set of goal states. A solution for *P* is a plan π = {*a*_1_, …, *a*_*k*_} of length *k* that modifies *s*_0_ into *G* by execution of actions *a*_1_, …, *a*_*k*_. The effect of executing a plan is defined by calling γ recursively: γ(…γ(γ(*s*_0_, *a*_1_), *a*_2_)…, *a*_*k*_) = *G*.

The Intervention Problem requires domain models that are distinct for the user, observer, and competitor. Let us define the user's domain model as *D*_user_ = (*F*_user_, *A*_user_, *s*_0_). The competitor can see the effects of the user but cannot take user actions. We denote the domain model for the competitor as *D*_other_ = (*F*_user_ ∪ *F*_other_, *A*_other_, *s*_0_). Although the observer can see the actions of the user and the competitor, it does not execute actions. This is because we are only focused on the recognition aspect of the Intervention Problem. So the observer's domain model is *D*_observer_ = (*F*_user_ ∪ *F*_other_, *A*_user_ ∪ *A*_other_, *s*_0_).

### 3.3. The Unsafe Intervention Suffix and Direct/Indirect Contributors

As seen in Section 2, when presented with an action, the observer must intervene after analyzing the remaining plans considering u and u ∪ d. The Intervention Suffix analysis allows the observer to identify the observations that help the user avoid u.

**Definition 1 (Intervention Suffix)**. *Let a_k_ be an action that achieves some goal g from state s_k−1_ (i.e., γ(s_k−1_, a_k_) = g). An Intervention Suffix X_g_ = (a_1_, a_2_, …, g) is a sequence of actions that starts in a_1_ and ends at g* ⊂ {u, u ∪ d}.

Suppose that we want to determine a path to u where the Intervention Suffix is *X*_u_ = (*o*_*i*_, …, u). By replacing *g* with u, or u ∪ d, an automated planner can be used to generate an Intervention Suffix. We use the set of Intervention Suffixes ($X_{◇}$), where $X_{\text{u}},X_{\text{u}\cup \text{d}}\in X_{◇}$ generated by the Top-K planner (Riabov et al., 2014) to evaluate Unsafe Suffix Intervention in Section 5. We refer to a single suffix (i.e., plan) leading to u as π_u_ and a set of such plans as Π_u_. Similarly, we refer to suffixes leading to d and avoids u as π_d_ and a set of such plans as Π_d_.

Actions may directly or indirectly contribute to u. The direct and indirect contributors express different degrees of urgency to intervene. A directly contributing action indicates that u is imminent and intervention must happen immediately. An indirectly contributing sequence indicates that u is not imminent, but intervention may still be an appropriate decision. Next, we define directly contributing actions and indirectly contributing sequences.

**Definition 2 (Direct Contributor)**. A directly contributing action *a_crit_ occurs in an undesirable plan π_u_ ∈ Π_u_ and execution of a_crit_ in state s results in a state s′ such that γ*(*s, a*_*crit*_) = u.

**Example of a directly contributing action**. In the example illustrated in Figure 2B, d = {(ON T A)(ON A D)}(i.e., TAD) and u = {(ON B A)(ON A D)} (i.e., BAD). The user may enable d when he reveals {(STACK USER A D)}, but at the same time this create an opportunity for the competitor to reach u first. However, the observer flags {(STACK COMPETITOR B A)} for intervention (marked *Yes*) because the post-condition of STACK B A satisfies u. Therefore, STACK COMPETITOR B A is a *directly contributing action*.

**Definition 3 (Indirect Contributor)**. An indirectly contributing sequence *q*_*crit*_ *is a totally ordered action sequence in an undesirable plan in* Π_u_ *and the first action in* *q*_*crit*_ *is equal to the first action in the Intervention Suffix* *x*_1_ ∈ *X*_u ∪ d_. *Executing actions in* *q*_*crit*_ *from state* *s* *results in a state* *s*′ *such that* γ(*s, q*_*crit*_) = {u ∪ d}.

**Example of an indirectly contributing sequence**. Figure 2A illustrates an *indirectly contributing sequence*. The totally ordered sequence {STACK COMPETITOR T P, STACK USER U T, PICKUP USER C, STACK USER C U} is an *indirectly contributing sequence* because the actions in the sequence together satisfies u ∪ d. Any Intervention Suffix *X*_*g*_ containing actions from an indirectly contributing sequence must be flagged for intervention.

We next formally define the Unsafe Intervention Suffix *X*_unsafe_.

**Definition 4 (Unsafe Suffix)**. *An Intervention Suffix X of length k is unsafe if there is at least one action x_i_* ∈ *X* (1 ≤ *i* ≤ |*X*|) *such that x_i_ is a directly contributing action or x_i_ is in a indirectly contributing sequence*.

In the example in Figure 2B, *X*_unsafe_ = (PICKUP USER A, STACK USER A D, PICKUP COMPETITOR B, PICK USER UP T, STACK COMPETITOR B A, u) because of the directly contributing action STACK COMPETITOR B A. In the example in Figure 2A, *X*_unsafe_ = (PICKUP USER U, PICKUP COMPETITOR T, STACK COMPETITOR T P, STACK COMPETITOR T P, STACK USER U T, PICKUP USER C, STACK USER C U, u ∪ d) because it contains the actions from an indirectly contributing sequence.

### 3.4. The Family of Intervention Problems

We now define a general form of the Intervention Problem. Let *plan*(*o*_*i*_, *g*) be some general method to generate suffixes for π_d_ and π_u_; in Section 4.2 we will show how we can use classical planning.

**Definition 5 (Intervention Problem)**. *Let* $I=\left(D,\text{d},\text{u},H,o_{i},X_{◇}\right)$ *be a tuple where* *D* = 〈*F, A, s*_0_〉 *is a planning domain, d* ⊂ *F* *is a desirable state, u* ⊂ *F* *is an undesirable state*, *H* = (*o*_1_, *o*_2_, …, *o*_*i*−1_) *is a history of previously observed actions*, *o*_*i*_ *is the* *presented action* *that the user would like to perform, and* $X_{◇}=\left\{X_{j}=plan\left(o_{i},g\right)|\forall g\in \left\{\text{u},\text{u}\cup \text{d}\right\}\right\}$ *for* *j* ≥ 0 *is a set of suffixes leading to* u *and* u ∪ d. *The* Intervention Problem *is a function* intervene(I):I→{No,Yes} *that determines for the presented action* *o*_*i*_ *whether to intervene*.

To decide whether $X_{◇}$ contains an unsafe suffix, the observer analyzes suffixes for *plan*(*o*_*i*_, u), and *plan*(*o*_*i*_, u ∪ d). If the observer finds that $X_{◇}$ contains an unsafe suffix then *o*_*i*_ is not accepted into *H*. Let history *H* = (*o*_1_[*s*_1_], *o*_2_[*s*_2_], …, *o*_*i*−1_[*s*_*H*_]) be a sequence of previously observed actions, which started from *s*_0_ with the implied resulting states in brackets. The state resulting from applying history to *s*_0_ is *s*_*H*_ = γ(*s*_0_, *H*). If *o*_*i*_ is accepted, then $H^{\prime }=\left\{H\cup o_{i}\right\}$ and the effect of *o*_*i*_ is represented in state as defined by γ(*s*_*H*_, *o*_*i*_). A solution to I is sequence of {*No, Yes*} decisions for each step *i* of observations. We next explore special cases of the Intervention Problem, namely single-user intervention, competitive intervention, and the most general form of multi-agent intervention.

#### 3.4.1. Intervention for a Single User

When only the user and the observer are present, the user solves the planning problem *P*_user_ = 〈*F*_user_, *A*_user_, *s*_0_, d〉, and incrementally reveals it to the observer. At each point in the plan solving *P*_user_, the observer must analyze $I=\left(D_{\text{user}},\text{d},\text{u},H,o_{i},X_{◇}\right)$, where $X_{◇}$ is generated in some sensible way.

#### 3.4.2. Intervention in the Presence of a Competitor

If a competitor is present, the user's planning problem *P*_user_ is the same as before. However, the competitor also solves a planning problem *P*_other_ = 〈*F*_user_ ∪ *F*_other_, *A*_other_, *s*_0_, u〉. Note that the competitor has a limited set of actions in *A*_other_ to create states that will lead to u and *A*_other_ ∩ *A*_user_ = ∅. The user's and the competitors solutions to *P*_user_ and *P*_other_ are revealed incrementally. Therefore, $H,X_{◇}\subset \left\{A_{\text{user}}\cup A_{\text{other}}\right\}$. To decide whether *X* $\in X_{◇}$ is unsafe, the observer analyzes $I=\left(D_{\text{other}},\text{d},\text{u},H,o_{i},X_{◇}\right)$, where *D*_other_ = 〈*F*_user_ ∪ *F*_other_, *A*_other_ ∪ *A*_user_, *s*_0_〉. The observer accepts *o*_*i*_ into *H* as before.

#### 3.4.3. Human-Aware Intervention

When performing tasks with a steep learning curve (e.g., a puzzle, problem solving), human users may initially make more mistakes or explore different (sub-optimal) solution strategies. Over time, a human may learn to make better choices that result in more efficient plans. Because of the inconsistencies in solution search strategy, we cannot accurately project the goals of the human user. Learning properties about the history *H* will help the observer recognize when the user is about to make a mistake and use that information to guide the search task on behalf of the user. When humans are solving problems in real time, the criteria for intervention may place more emphasis on the history *H* than on the suffixes $X_{◇}$. In Section 6, we consider the special case where $X_{◇}=\emptyset$.

## 4. Recognizing Unsafe Suffixes

We present two solutions for recognizing unsafe suffixes. Recall that in Definition 5, the observer's decision space $X_{◇}$ is derived such that $X_{◇}=\left\{X_{j}=plan\left(o_{i},g\right)|\forall g\in \left\{\text{u},\text{u}\cup \text{d}\right\}\right\}$ for *j*≥0. In the first solution, we implement the function *plan*(*o*_*i*_, *g*) as an Intervention Graph (in Section 4.1 and Section 4.2). In the second solution, we implement *plan*(*o*_*i*_, *g*) by sampling the plan space using an automated planner and use plan distance metrics to make the decision about whether to intervene (Section 4.3).

### 4.1. The Intervention Graph

The Intervention Graph models the decision space of the observer for the Intervention Problem I. We can extract several features from the Intervention Graph to derive functions that map the presented observation *o*_*i*_ to intervention decisions. The Intervention Graph captures where u, and u ∪ d lie in the projected state space from *s*_*H*_. We can use properties of the graph to evaluate how close the current projection *H* is to u and u ∪ d and identify directly and indirectly contributing actions.

The Intervention Graph consists of alternating state and action layers where each state layer consists of predicates that have been made true by the actions in the previous layer. The root node of the tree is *s*_*H*_. An action layer consists of actions (defined in *D*_user_ or *D*_other_) whose preconditions are satisfied in the state from the previous layer. Algorithm 1 describes the process of building the Intervention Graph. The algorithm takes as input a domain theory *D* (for *D*_user_ or *D*_other_), *s*_*H*_ and *g* = {u, u ∪ d} (lines 1-2). When *H* = ∅, the root of the tree is set to *s*_0_. Next, using the domain theory, actions whose preconditions are satisfied at current state are added to the graph (lines 5-6). Each action in level *i* spawns possible states for level *i* + 1. Line 7 ensures that the actions that immediately inverts the previous action are not added to the graph. For each resulting state a search node is created, with an edge representing the action responsible for the state transition (lines 8-10). The method is executed recursively for each open search node until d and u are added to the graph generates $X_{◇}$ for the observer (line 11). To ensure that only realistic plans are explored, we do not add no-op actions to the action layers in the graph. When the user and the competitor present new actions, the root of the graph is changed to reflect the new state *s*_*H*_ and subsequent layers are modified to that effect.

**Algorithm 1:** Build Intervention Graph

**Require**: *D*, *s*_*H*_, *g*
1: *i* = 0; *s*_*i*_ ← *s*_0_
2: **procedure** expandgraph(*D, s*_*H*_, *g*)
3: **if** *s*_*i*_ ⊧ *g* **then** return 〈*V, E*〉
4: **else**
5: **for** action *a* where *Pre*(*a*) ∈ *s*_*i*_ **do**
6: *s*_*i*+1_ ← ((*s*_*i*_ \ *Del*(*a*)) ∪ *Add*(*a*))
7: **if** *s*_*i*+1_ ≡ *s*_*i*_ **then** continue
8: **end if**
9: *v* ← AddVertex (*s*_*i*+1_)
10: *e* ← AddEdge (*s*_*i*_, *s*_*i*+1_, *a*)
11: *V* ∪ {*v*}; *E* ∪ {*e*}
12: ExpandGraph (*D, s*_*i*+1_, *g*)
13: **end for**
14: **end if**
15: **end procedure**

The Intervention Graph is a weighted, single-root, directed acyclic connected graph *IG* = 〈*V, E*〉, where *V* is the set of vertices denoting possible states the user could be in leading to *g*, and *E* is the set of edges representing actions from *D*_user_ or *D*_other_ depending on single user intervention or competitive intervention. *X*_unsafe_ is a path from the root of the *IG* to u ∪ d or u. In contrast, a safe suffix *X*_safe_ is a path from the root of the *IG* to d and avoids u.

### 4.2. Intervention Graph Features

**Figure 3:**
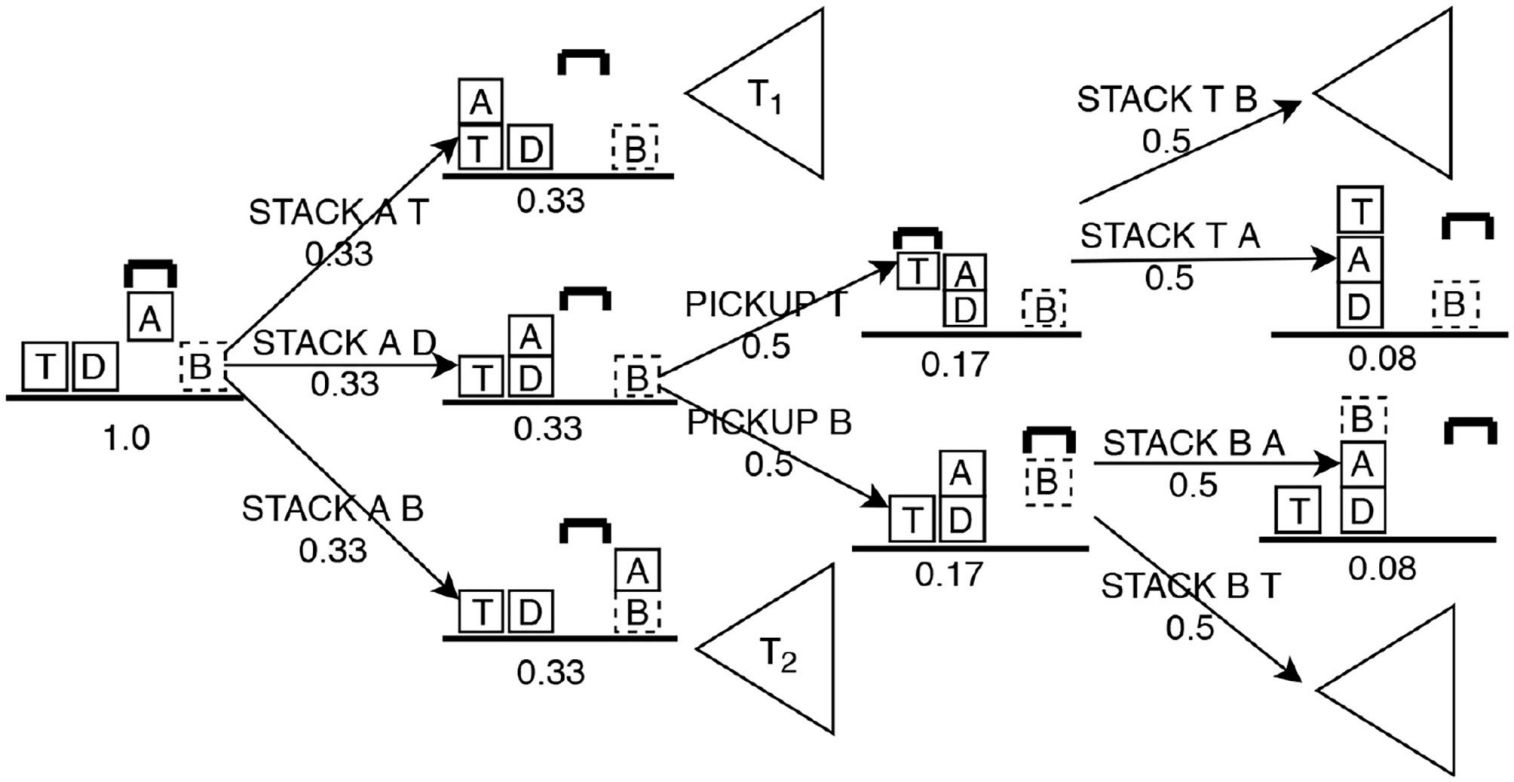
Fragment of the decision space after PICKUP A has been proposed for block-words example in Figure 2B. Numbers under each state and action indicate the probability. Sub trees *T*_1_ and *T*_2_ are not expanded for simplicity.

We extract a set of features from the Intervention Graph that help determine whether to intervene. These features include: Risk, Desirability, Distance to d, Distance to u and Percentage of active undesirable landmarks. We use these features to train a classifier that learns to identify actions in *a*_*crit*_ and *q*_*crit*_. Figure 3 illustrates a fragment of the Intervention Graph from Figure 2B after the user presents the action PICK-UP A, which we will use as a running example to discuss feature computation.

#### 4.2.1. Risk (*R*)

Risk quantifies the probability that the presented action will lead to u. We model the uncertainty the observer has about the next action as a uniform probability distribution across the set of applicable actions whose preconditions are satisfied in current state. We define risk *R* as the posterior probability of reaching u while the user is trying to achieve d. We extract $X_{◇}$ from the Intervention Graph by searching breadth-first from the root until vertices containing d is found, including the paths in which the user has been subverted to reach u. By construction, d will always be a leaf. Let $|X_{◇}|=n$. The set of unsafe intervention suffixes, $X_{■}$ is such that $X_{■}\subseteq X_{◇}$ and $|X_{■}|=m$ and (*m* ≤ *n*). We compute posterior probability of reaching u for $X_{■}$, using the chain rule in probability as, $Pr_{\text{unsafe}}=\prod_{i=k}^{1}P\left(\alpha_{i}|\alpha_{i-1}\ldots \alpha_{1}\right)$, and α_*j*_ ∈ {*A*_user_} or α_*j*_ ∈ {*A*_user_ ∪ *A*_other_} and *k* is the length of the suffix until u is reached. Then:


$$\begin{array}{l} R=\{\begin{array}{cc} \frac{\sum_{i=1}^{m}Pr_{{\text{unsafe}}_{i}}}{m} & m>0\\ 0 & m=0 \end{array} \end{array}$$

In Figure 3, (*n* = 6) and (*m* = 1). Since we assumed full observability for the observer, the root of the tree (current state) is assigned the probability of 1.0. Actions that are immediately possible after the current state are each assigned probabilities following a uniform distribution across the branching factor (0.33). Then for each applicable action in the current state, the resulting state gets the probability of (1.0 × 0.33 = 0.33). Similarly, we apply the chain rule of probability for each following state and action level in the graph until u first appears in the suffix. $R=\frac{0.08}{1}=0.08$.

#### 4.2.2. Desirability (*D*)

Desirability measures the effect of the observed action to help the user pursue d safely. Let $X_{□}$ be the set of suffixes that reach d while avoiding u. Then $X_{□}=X_{◇}\backslash X_{■}$. We compute posterior probability of reaching d avoiding u for $X_{□}$, using the chain rule in probability as, $Pr_{safe}=\prod_{i=k}^{1}P\left(\alpha_{i}|\alpha_{i-1}\ldots \alpha_{1}\right)$, and α_*j*_ ∈ {*A*_user_} or α_*j*_ ∈ {*A*_user_ ∪ *A*_other_} and *k* is the length of path. Then:


$$\begin{array}{l} D=\{\begin{array}{cc} \frac{\sum_{i=1}^{\left|X_{□}\right|}Pr_{safe_{i}}}{\left|X_{□}\right|} & \left|X_{□}\right|>0\\ 0 & \left|X_{□}\right|=0 \end{array} \end{array}$$

In Figure 3, there are five paths where user achieved d without reaching u (two in subtree *T*_1_, three in the expanded branch). Following the same approach to assign probabilities for states and actions, $D=\frac{\left(0.08+0.08+0.08+0.04+0.04\right)}{5}=0.07$. *R* and *D* are based on probabilities indicating the confidence the observer has about the next observation.

#### 4.2.3. Distance to u (δ_u_)

This feature measures the distance to state u from the current state in terms of the number of edges in the paths in $X_{◇}$ extracted from the Intervention Graph. We extract $X_{◇}$ from the Intervention Graph from the root to any vertex containing d, including the paths in which the user has been subverted to reach u instead. Let $|X_{◇}|=n$. The set of suffixes that reach u, $X_{■}$ is such that $X_{■}\subseteq X_{◇}$ and $|X_{■}|=m$ and (*m* ≤ *n*). We count *s*, the number of the edges (actions) before u is reached for each path in $X_{■}$ and δ_u_ is defined as the average of these distance values:


$$\begin{array}{l} \delta_{\text{u}}=\{\begin{array}{cc} \frac{\sum_{i=1}^{m}s_{i}}{m} & m>0\\ -1 & m=0 \end{array} \end{array}$$

In this formula, −1 indicates that the undesirable state is not reachable from the current state. For the example problem illustrated in Figure 3, $\delta_{\text{u}}=\frac{3}{1}=3$.

#### 4.2.4. Distance to d (δ_d_)

This feature measures the distance to d from current state. The path set $X_{□}$ contains action sequences that reach d without reaching u. We count *t*, the number of the edges where d is achieved safely for a path in $X_{□}$. Then, δ_d_ is defined as the average of these distances given by the formula:


$$\begin{array}{l} \delta_{\text{d}}=\{\begin{array}{cc} \frac{\sum_{i=1}^{\left|X_{□}\right|}t_{i}}{\left|X_{□}\right|} & \left|X_{□}\right|>0\\ -1 & \left|X_{□}\right|=0 \end{array} \end{array}$$

In this formula, −1 indicates that d cannot be reached safely from the current state. For the example problem illustrated in Figure 3, $\delta_{\text{d}}=\left\lceil \frac{3+3+7+7+3}{5}\right\rceil =5$.

#### 4.2.5. Active Attack Landmark Percentage ($L_{ac}$)

This feature captures the criticality of the current state toward contributing to u. We used the algorithm proposed by Hoffmann et al. (2004) to extract fact landmarks for the planning problem *P* = 〈*D*_other_, u〉 or *P* = 〈*D*_user_, u〉. *Landmarks* have been successfully used in deriving heuristics in Plan Recognition (Vered et al., 2018) and generating alternative plans (Bryce, 2014). We define *attack landmarks* ($L_{u}$) to be those predicates which must be true to reach u. We compute the percentage of active attack landmarks in the current state ($L_{ac}$), where $L_{ac}=\frac{l}{\left|L_{u}\right|}$. In Figure 3, *l* = 4 and $L_{ac}=4/10=0.4$. For each presented action, the Intervention Graph is generated and features are computed, producing the corresponding feature vector. Landmarks are computed apriori.

### 4.3. Plan Space Sampling and Plan Distance Metrics

Extracting Intervention Graph features can be intractable when the graph is large. Therefore, for our second method for implementing *plan*(*o*_*i*_, *g*) we define an additional set of features, called *Sampled Features* by sampling the plan space for the observer. If the Intervention Problem is defined for a single user, we sample the observer's plan space by using an automated planner to find solutions for *P*_observer_ = 〈*F*_user_, *A*_user_, *s*_0_, *g*〉, where *g* ∈ {d, u}. If the Intervention Problem is defined for a user and a competitor, we sample the observer's plan space by using an automated planner to find solutions for *P*_observer_ = 〈*F*_user_ ∪ *F*_other_, *A*_user_ ∪ *A*_other_, *s*_0_, *g*〉, where *g* ∈ {d, u}. Note that in this method we omit u ∪ d for generating plans. Sampled plans are generated with the Top-K planner (Riabov et al., 2014). We estimate the Risk and Desirability using plan distance metrics. The intuition is that if the actor is executing an unsafe plan, then that plan should be more similar to a sample of unsafe plans, compared to a sample of safe plans.

For each presented action, the observer computes plan distances between a reference plan (π′) and sampled plans (Π^′′^) for both u and d. We generate the observation compatible plan by concatenating the observation history with the optimal plan that reaches u (and d) to produce π′ (see Vered and Kaminka, 2017).

We use the Top-K planner with K=50 to sample the plan space. We use Action Set Distance (ASD), State Sequence Distance (SSD), Causal Link Distance (CLD) (Nguyen et al., 2012), Generalized Edit Distance (GED) for sequences of states and actions (Sohrabi et al., 2016b) to measure the distances between the reference plan and the sampled plans for d and u. When an action is presented, π′ is computed. Then, observation compatible Top-K plans are produced for u and d separately. The medians of ASD, CLD and SSD, minimum remaining actions to u and d, minimum action GED and state GED are computed for u and d for all 〈reference, sample〉 pairs. Finally, we also compute the Landmark Completion Heuristic proposed by Pereira et al. (2017). This produces the Sampled Feature vector for the presented action. Algorithm 2 shows the pseudo-code for computing the Sampled Feature vector.

**Algorithm 2:** Build Sampled Feature Vector

**Require**: *D*, *s*, u, d
1: *i* = 0; *s*_*i*_ ← *s*_0_
2: $prefix,suffix,\Pi^{\prime \prime },V\leftarrow \emptyset$
3: **procedure** sampledsuffixes(*D*_1_, *s*, u, d, *O*)
4: **for** *o* ∈ *O* **do**
5: *prefix* ← *prefix* + *o*
6: *s*_*i*+1_ ← ((*s*_*i*_ \ *Del*(*o*)) ∪ *Add*(*o*))
7: **for** *g* ∈ {u, d} **do**
8: *suffix* ← *OptimalPlan*(*s, g*)
9: π′ ← *prefix* + *suffix*
10: Π^′′^ ← Observation compatible Top-K plans for*g*
11: *v*_1_ ← MedianActionSetDist(π′, Π^′′^)
12: *v*_2_ ← MedianCausalLinkDist(π′, Π^′′^)
13: *v*_3_ ← MedianStateSequenceDist(π′, Π^′′^)
14: *v*_4_ ← MinimumRemainingDistToState (*g*, Π^′′^)
15: *v*_5_ ← MinimumActionGED (π′, Π^′′^)
16: *v*_6_ ← MinimumStateGED (π′, Π^′′^)
17: $V\left(o\right)\leftarrow \left[v_{1},v_{2},v_{3},v_{4},v_{5},v_{6}\right]$
18: **end for**
19: *v*_7_ ← ComputeLandmarkCompletionHeuristic (u)
20: $V\left(o\right)\leftarrow V\left(o\right)+\left\{v_{7}\right\}$
21: **end for**
22: **end procedure**

### 4.4. Learning Intervention

We train a classifier to categorize the presented observation *o*_*i*_ into two classes: (Yes) indicating intervention is required and (No) indicating otherwise. We chose Naive Bayes, K-nearest neighbors, decision tree and logistic regression classifiers from Weka^1^. Given observations labeled as Yes/No and corresponding feature vectors as training examples, we train the classifiers with 10-fold cross validation. The trained model is used to predict intervention for previously unseen Intervention Problems. Attribute selected classifiers filter the feature vector to only select critical features. This step reduces complexity of the model, makes the outcome of the model easier to interpret, and reduces over-fitting.

We generated training data from 20 Intervention Problems using the benchmark domains. We used the Blocks World domain to model competitive Intervention Problems and Ferry, EasyIPC and Navigator domains to model Standard Intervention Problems. We restricted the number of observation traces per Intervention Problem to 100 for training the classifiers.

The decision tree classifier is tuned to pruning confidence = 0.25 and minimum number of instance per leaf = 2. The K-nearest neighbor classifier is tuned to use k = 1 and distance measure = Euclidean. The logistic regression classifier is tuned for ridge parameter = 1.0E-8. The Naive Bayes classifier is tuned with the supervised discretization = True.

## 5. Evaluating Intervention Recognition

We compare the learning based intervention accuracy to three state-of-the-art Plan Recognition algorithms from the literature. Our evaluation focuses on two questions: (1) Using domain-independent features indicative of the likelihood to reach u from current state, can the observer correctly recognize directly and indirectly contributing suffixes to prevent the user from reaching u? and (2) How does the learning approach perform against state-of the-art Plan Recognition? To address the first question, we evaluated the performance of the learned model on unseen Intervention Problems.

The benchmarks consist of Blocks-words, IPC Grid, Navigator and Ferry domains. For the **Blocks-words** domain, we chose word building problems. The user and the competitor want to build different words with some common letters. The problems in **Blocks-1** model intervention by identifying the direct contributors (*a*_*crit*_), whereas the problems in **Blocks-2** model intervention by identifying the indirect contributors (*q*_*crit*_) in the Blocks-words domain. In the **IPC grid** domain, the user moves through a grid to get from point A to B. Certain locked positions on the grid can be opened by picking up keys. In the **Navigator** domain, the user moves from one point on a grid to another. In IPC Grid and Navigator domains, we designated certain locations on the grid as traps. The goal of the user is to navigate to a specific point on the grid without passing through the trap. In the **Ferry** domain, a single ferry moves cars between different locations. The ferry's objective is to transport cars to specified locations without using a port, which has been *compromised*.

To evaluate our trained classifiers, we generate 3 separate test problem sets with 20 problems in each set (total of 60) for the benchmark domains. The test problems differ from the training data. The three test problems vary the number of blocks in the Blocks Words domain, size of the grid (Navigator, IPC-Grid), accessible and inaccessible paths on the grid (Navigator, IPC-Grid), and properties of the artifacts in the grid (IPC-Grid). Each test problem includes 10 observation traces (total of 600 test cases). We designed the Blocks-1, IPC-Grid, Ferry, and Navigator problems specifically considering desirable/undesirable goal pairs that are difficult to disambiguate using existing plan recognition algorithms. We designed the problems in Blocks-2 domain to include problems that will be easier to solve by existing plan recognition algorithms.

We define true-positive as the classifier correctly identifying the presented action to be in *a*_*crit*_ or *q*_*crit*_. True-negative is an instance where the classifier correctly identifying an action as not belonging to *a*_*crit*_ or *q*_*crit*_. False-positives are instances where classifier incorrectly identifies an action as belonging to *a*_*crit*_ or *q*_*crit*_. False-negatives are instances where the classifier incorrectly identifies the presented action as not belonging to *a*_*crit*_ or *q*_*crit*_. Naturally, our test observation traces contain a large number of negatives. To offset the bias introduced to the classifier by the class imbalance, we report Matthews correlation coefficient (MCC) because it gives an accurate measure of the quality of a binary classification while taking into account the different class sizes. We also report the F-score $=\frac{tp}{tp+1/2\left(fp+fn\right)}$ for the classifiers, where *tp*, *fp*, *fn* are the number of true positives, false positives and false negatives, respectively.

We implemented three state-of-the art Plan Recognition algorithms to compare the accuracy of intervening by Plan Recognition to the proposed learning based intervention. We selected Ramirez and Geffener's probabilistic Plan Recognition algorithm (Ramırez and Geffner, 2010) (both the satisficing, and optimal implementations) and the Goal Recognition with Goal Mirroring algorithm (Vered et al., 2018). To generate satisficing plans we used the Fast Downward planner with the FF heuristic and context-enhanced additive heuristic (Helmert, 2006). To generate the optimal cost plans we used the HSP planner (Bonet and Geffner, 2001). For each presented action, the observer solves a Plan Recognition problem using each approach. We assumed uniform priors for over u and d. If u is the top ranked goal for the presented action, then it is flagged as requiring intervention. The assumption is that these algorithms must also be able to correctly identify u as the most likely goal for the actions in *a*_*crit*_ and *q*_*crit*_. We used the same test data to evaluate accuracy.

Tables 3 and 4 shows that the classifiers trained with features from the Intervention Graph achieve high accuracy for all the domains when predicting intervention for both identifying actions in *a*_*crit*_ and *q*_*crit*_. The MCC value shows that the imbalance in class sizes does not bias the classifier. Low false positives and false negatives suggest that the user will not be unnecessarily interrupted. As expected performance degrades when we use a sampled plan space to derive features. However, the features derived from sampling the plan space produce equally good classifiers compared to the intervention graph method, when modeling intervention by identifying actions in *a*_*crit*_ and *q*_*crit*_ for the Blocks-word problems. For the Intervention Problems modeled using the benchmark domains, we were able to find that at least one of the selected classifiers responded with very high F-score when trained using plan similarity features. The exception to this pattern was the Intervention Problems modeled using the Ferry domain.

**Table 3:** F-score and MCC for predicting intervention using Intervention Graph and the Plan Space Sampling methods for Naive Bayes and Decision Tree classifiers.

**Domain**	**Naive bayes**						**Decision tree**					
	**Test set 1**		**Test set 2**		**Test set 3**		**Test set 1**		**Test set 2**		**Test set 3**	
	**F-score**	**MCC**	**F-score**	**MCC**	**F-score**	**MCC**	**F-score**	**MCC**	**F-score**	**MCC**	**F-score**	**MCC**
**Intervention graph method**												
Blocks-1	1	1	1	1	1	1	1	1	1	1	1	1
Blocks-2	1	1	1	1	1	1	1	1	1	1	1	1
EasyIPC	1	1	1	1	1	1	1	1	1	1	1	1
Ferry	1	1	1	1	1	1	1	1	1	1	1	1
Navigator	1	1	1	1	0.99	0.99	0.87	0.87	0.72	0.74	0.90	0.90
**Plan space sampling method**												
Blocks-1	0.25	0.33	0.25	0.33	0.25	0.33	0.25	0.33	0.25	0.33	0.25	0.33
Blocks-2	1	1	1	1	1	1	1	1	0.99	0.99	1	1
EasyIPC	1	1	1	1	1	1	1	1	1	1	1	1
Ferry	0.34	0.33	0.32	0.31	0.02	–0.004	0.25	0.28	0.24	0.23	0.86	0.86
Navigator	1	1	1	1	1	1	0.62	0.65	1	1	1	1

**Table 4:** F-score and MCC for predicting intervention using Intervention Graph and Plan Space Sampling methods for logistic regression and k-nearest neighbor classifiers.

**Domain**	**Logistic regression**						**K-nearest**					
	**Test set 1**		**Test set 2**		**Test set 3**		**Test set 1**		**Test set 2**		**Test set 3**	
	**F-score**	**MCC**	**F-score**	**MCC**	**F-score**	**MCC**	**F-score**	**MCC**	**F-score**	**MCC**	**F-score**	**MCC**
**Intervention graph method**												
Blocks-1	1	1	1	1	1	1	1	1	1	1	1	1
Blocks-2	1	1	1	1	1	1	1	1	1	1	1	1
EasyIPC	0.88	0.87	0.88	0.87	0.86	0.86	1	1	1	1	1	1
Ferry	1	1	1	1	1	1	1	1	1	1	1	1
Navigator	1	1	1	1	0.99	0.99	1	1	0.96	0.96	0.99	0.99
**Plan space sampling method**												
Blocks-1	0.25	0.33	0.25	0.33	0.25	0.33	1	1	1	1	1	1
Blocks-2	1	1	1	1	1	1	1	1	1	1	1	1
EasyIPC	0.64	0.63	0.46	0.44	0.67	0.66	0.05	–0.04	0.04	–0.03	0.05	–0.02
Ferry	0.31	0.32	0.23	0.22	1	1	0.33	0.40	0.13	0.15	0.81	0.82
Navigator	0.60	0.59	0.98	0.94	0.97	0.97	0.61	0.65	1	1	1	1

Features derived from the Intervention Graph accurately recognize actions in *a*_*crit*_ and *q*_*crit*_ where the user has limited options available to reach the desirable goal while avoiding the undesirable state. Thus the classifiers perform well in recognizing critical actions in new problems. The sampled features rely on the learning algorithm to produce accurate results when predicting intervention.

Comparing the results in Table 5, learning methods outperform existing Plan Recognition algorithms when predicting intervention. The algorithms we selected clearly struggled to predict intervention in the Navigator domain producing many false positives and false negatives. These results suggest that although we can adopt existing Plan Recognition algorithms to identify when the user needs intervention, it also produces a lot of false negatives and false positives in correctly identifying which state (desirable/undesirable) is most likely given the observations, especially when the desirable and undesirable plans have a lot of overlap. Comparing recognition accuracy of Blocks-1 and Blocks-2 problems using plan recognition algorithms to intervene, we see that when the undesirable state develops over a long period of time, the plan recognition algorithms recognize the undesirable plan with high accuracy. In other words, if the d and u are sufficiently distinct and are far apart in the state space, intervention by using plan recognition algorithms are as effective as our proposed learning based intervention methods in most cases. When the desirable and undesirable states are closer, existing plan recognition algorithms produce many false positives/negatives.

**Table 5:** F-score and Matthews Correlation Coefficient (MCC) for recognizing intervention using probabilistic goal recognition (RG) algorithm (Ramırez and Geffner, 2010).

**Domain**	**Test set 1**						**Test set 2**						**Test set 3**					
	**RG (LAMA)**		**RG (HSP)**		**GM**		**RG (LAMA)**		**RG (HSP)**		**GM**		**RG (LAMA)**		**RG (HSP)**		**GM**	
	**F-score**	**MCC**	**F-score**	**MCC**	**F-score**	**MCC**	**F-score**	**MCC**	**F-score**	**MCC**	**F-score**	**MCC**	**F-score**	**MCC**	**F-score**	**MCC**	**F-score**	**MCC**
Blocks-1	0.38	0.45	0.38	0.45	0.36	0.43	0.43	0.49	0.43	0.49	0.39	0.45	0.40	0.47	0.40	0.47	0.38	0.45
Blocks-2	1	1	0.90	0.90	1	1	1	1	0.90	0.90	1	1	1	1	0.90	0.90	1	1
EasyIPC	0.13	0.05	0.13	0.05	0.10	0.01	0.21	0.17	0.18	0.13	0.12	0.06	0.23	0.19	0.22	0.19	0.14	0.09
Ferry	0.17	0.18	0.22	0.20	0.10	0.08	0.22	0.23	0.11	0.06	0.15	0.09	0.15	0.17	0.47	0.52	0.21	0.34

*RG (LAMA) is when a satisficing planner (LAMA) is used to generate the plans for probabilistic goal recognition. RG (HSP) is when an optimal (HSP) planner is used to generate the plans for probabilistic goal recognition. Goal mirroring (GM) implements the recognition algorithm proposed by Vered et al. (2018) and uses the JavaFF planner to generate the plans. For the Intervention problems in the Navigator domain, the true-negative, false negative rates were 100% each. Therefore, F-score and MCC are not reported*.

This is unhelpful specially considering human users because, when the intervening agent produces many false alarms and/or miss critical events that must be intervened, the human users may get frustrated and turn off intervention. Therefore, the learning approach is better suited for intervention because the observer can target specific critical actions and at the same time the user is given some freedom to pursue a desirable goal.

### 5.1. Processing Times

Because the Intervention problem is defined for online environments, we now report the processing time comparison among the two proposed learning based Intervention algorithms and intervention using existing plan recognition algorithms. The experiments were run in an Intel Core i7 CPU at 1.30GHz x 8 machine running on Ubuntu 20.04LTS. We compute two evaluation metrics for the processing time comparison. **The total processing time** (*Q*) is the CPU time in milliseconds taken to process all the 20 Intervention problems in a test set. **The mean processing time** ($\bar{Q}$) is the CPU time in milliseconds taken to return the intervention decision for one incremental observation reveal. It is given by the equation: $\bar{Q}=\frac{Q}{\text{number of observations in the test set}}$. Tables 6 and 7 show the *Q* and $\bar{Q}$ values for returning the intervention decisions using the classifiers trained with the Intervention Graph features and the plan distance features. When the classifiers are trained with the Intervention Graph features the smallest $\bar{Q}$ (< 70 ms) are reported for the Blocks-words domain. The largest $\bar{Q}$ values are reported for the EasyIPC and the Ferry domain (< 5.5 s). When the classifiers are trained with the plan distance features, $\bar{Q}$ is larger compared to the previous case. However, the values for $\bar{Q}$ are still lower (< 620 ms) compared to the other domains. The largest $\bar{Q}$ are reported for the EasyIPC and the Ferry domains (< 1.8 s). There is a large range of the total processing times (maximum *Q*- minimum *Q*) among the Intervention problems from different domains.

**Table 6:** Total processing time in seconds (*Q*) for all Intervention problems in set 1, 2, and 3 and the mean processing time in milliseconds for each incrementally revealed observation ($\bar{Q}$) for predicting intervention with Naive Bayes and Decision Tree classifiers using features from the Intervention Graph and the Plan Space Sampling methods.

**Domain**	**Naive bayes**						**Decision tree**					
	**Test set 1**		**Test set 2**		**Test set 3**		**Test set 1**		**Test set 2**		**Test set 3**	
	***Q* (s)**	**$\bar{Q}$ (ms)**	***Q* (s)**	**$\bar{Q}$ (ms)**	***Q* (s)**	**$\bar{Q}$ (ms)**	***Q* (s)**	**$\bar{Q}$ (ms)**	***Q* (s)**	**$\bar{Q}$ (ms)**	***Q* (s)**	**$\bar{Q}$ (ms)**
**Intervention graph method**												
Blocks-1	42.4	64	39.9	60	42.5	64	40.6	61	39.3	59	42.0	63
Blocks-2	40	60	38.1	57	38.4	58	40.4	61	38.0	57	40.2	60
EasyIPC	7519.9	3,484	14858.0	5,371	805.3	403	7615.4	3,528	15842.8	5,727	802.6	402
Ferry	7992.5	3748	72.1	119	10656.9	4,492	7921.4	3,715	62.0	103	107945.0	4,550
Navigator	1530.0	1,969	880.4	896	77.4	102	1694.4	2,180	882.2	898	84.6	111
**Plan space sampling method**												
Blocks-1	409.5	620	405.6	614	402.6	610	338.3	512	365.1	553	365.9	554
Blocks-2	357.6	541	353.5	534	350.5	531	392.7	595	401.4	606	3743.4	567
EasyIPC	2274.8	1,054	4607.1	1,665	2100.2	1,053	2324.1	1,076	5023.9	1,816	2064.8	1035
Ferry	1479.8	694	195.0	323	1451.0	611	1564.9	733	219.1	364	1697.8	715
Navigator	1131.7	1,456	1231.4	1,254	373.4	493	1050.6	1,352	1183.2	1,204	369.7	489

**Table 7:** Total processing time in seconds (*Q*) for all Intervention problems in set 1, 2, and 3 and the mean processing time in milliseconds for each incrementally revealed observation ($\bar{Q}$) for predicting intervention with logistic regression and k-nearest neighbor classifiers using features from the Intervention Graph and the Plan Space Sampling methods.

**Domain**	**Logistic regression**						**K-nearest**					
	**Test set 1**		**Test set 2**		**Test set 3**		**Test set 1**		**Test set 2**		**Test set 3**	
	***Q* (s)**	**$\bar{Q}$ (ms)**	***Q* (s)**	**$\bar{Q}$ (ms)**	***Q* (s)**	**$\bar{Q}$ (ms)**	***Q* (s)**	**$\bar{Q}$ (ms)**	***Q* (s)**	**$\bar{Q}$ (ms)**	***Q* (s)**	**$\bar{Q}$ (ms)**
**Intervention graph method**												
Blocks-1	40.3	61	38.8	58	42.4	64	40.8	61	39.8	60	41.4	62
Blocks-2	40.7	61	38.9	58	39.4	59	41.3	62	39.5	59	41.6	63
EasyIPC	6955.0	3,222	15309.2	5,534	920.8	461	7159.8	3,317	14821.0	5,358	837.0	419
Ferry	7586.2	3,558	54.3	90	9598.3	4,046	8116.9	3,807	57.7	95	10029.4	4,228
Navigator	1615.4	2,079	868.6	884	91.7	121	1441.7	1,855	881.9	898	93.2	123
**Plan space sampling method**												
Blocks-1	390.3	591	405.8	614	398.4	603	376.3	570	372.2	563	372.4	564
Blocks-2	345.4	523	342.1	516	338.1	512	388.8	589	372.2	562	355.2	538
EasyIPC	2286.7	1,059	4596.6	1,661	1898.5	952	2528.8	1,171	4702.0	1,699	1928.9	967
Ferry	1531.9	718	210.0	348	1562.0	658	1549.2	726	212.2	352	1574.0	663
Navigator	1095.7	1,410	1209.9	1232	380.4	503	1076.1	1,384	1208.6	1,230	377.0	498

In contrast, Table 8 shows that when using plan recognition algorithms for intervention, $\bar{Q}$ is smaller compared to the $\bar{Q}$ in learning based intervention. For the Test Set 1, the smallest $\bar{Q}$ is reported for the EasyIPC problems when using probabilistic plan recognition using the optimal plans (< 42 ms). The largest $\bar{Q}$ is reported for the Ferry domain when using probabilistic plan recognition using the optimal plans (1.5 s), also in Test Set 1. Most of $\bar{Q}$ values are less than 232 ms. The range of the total processing times (maximum *Q*- minimum *Q*) among the Intervention problems from different domains is smaller compared to the range of the total processing times using the learning based intervention. While plan recognition algorithms *sometimes* return an intervention decision more quickly than the learning based intervention, their accuracy, precision, and recall is substantially lower than learning based intervention.

**Table 8:** Total processing time in seconds (*Q*) for all Intervention problems in set 1, 2, and 3 and the mean processing time in milliseconds for each incrementally revealed observation ($\bar{Q}$) for recognizing undesirable states with the algorithms: probabilistic goal recognition using satisficing (RG-LAMA) and optimal (RG-HSP) plans and goal mirroring (GM) using the JavaFF planner.

**Domain**	**Test set 1**						**Test set 2**						**Test set 3**					
	**RG (LAMA)**		**RG (HSP)**		**GM**		**RG (LAMA)**		**RG (HSP)**		**GM**		**RG (LAMA)**		**RG (HSP)**		**GM**	
	***Q* (s)**	**$\bar{Q}$ (ms)**	***Q* (s)**	**$\bar{Q}$ (ms)**	***Q* (s)**	**$\bar{Q}$ (ms)**	***Q* (s)**	**$\bar{Q}$ (ms)**	***Q* (s)**	**$\bar{Q}$ (ms)**	***Q* (s)**	**$\bar{Q}$ (ms)**	***Q* (s)**	**$\bar{Q}$ (ms)**	***Q* (s)**	**$\bar{Q}$ (ms)**	***Q* (s)**	**$\bar{Q}$ (ms)**
Blocks-1	147.9	224	65.8	99	151.6	229	147.1	222	60.1	91	148.9	225	148.1	224	63.3	95	152.0	230
Blocks-2	145.6	220	108.9	164	153.6	232	144.5	218	113.2	171	155.0	234	145.6	220	107.5	162	144.9	219
EasyIPC	474.0	219	91.5	42	450.6	208	599.5	216	418.3	151	549.0	198	427.2	214	70.6	35	407.9	204
Ferry	440.1	206	3266.6	1532	366.3	171	117.2	194	75.4	125	97.5	162	480.5	202	344.1	145	407.0	171

## 6. Human-Aware Intervention

The Human-aware Intervention Problem is a variant of the single-user intervention, so the history *H* consists of only accepted user actions. However, as mentioned in Section 3.2, the set of projections will be empty, that is $X_{◇}=\emptyset$ because planning is less accurate for projecting what the human user may do. This means the observer must emphasize analysis of *H*. Instead of relying on projections in $X_{◇}$, the observer *learns* the function *intervene*_*i*_. That is, at the presented action *o*_*i*_ it considers only *H*, d, and u and uses a trained machine learning algorithm to decide whether to intervene.

We introduce the Rush Hour puzzle as a benchmark in order to study how human users solve the puzzle as a planning task that requires intervention. We begin with the formal definitions of the Rush Hour puzzle. Next, we translate the Rush Hour problem into a STRIPS planning task and through a human subject study, we allow human users solve the planning problem and collect observation traces. We formally define the Human-aware Intervention problem and propose a solution that uses machine learning to learn properties of *H* to determine whether or not intervention is required. The observer for Human-aware Intervention should offer different levels of freedom to the user. At the lowest level of freedom, the observer will intervene just before the undesirable state. At increased levels of freedom, the observer offers the user enough time to recover from the undesirable situation. Varying the level of freedom allows the observer to gradually guide the user toward d without directly giving away the complete solution. We evalute the accuracy of our learning approach using the observation traces collected from the human subject study.

### 6.1. The Rush Hour Puzzle

**Figure 4:**
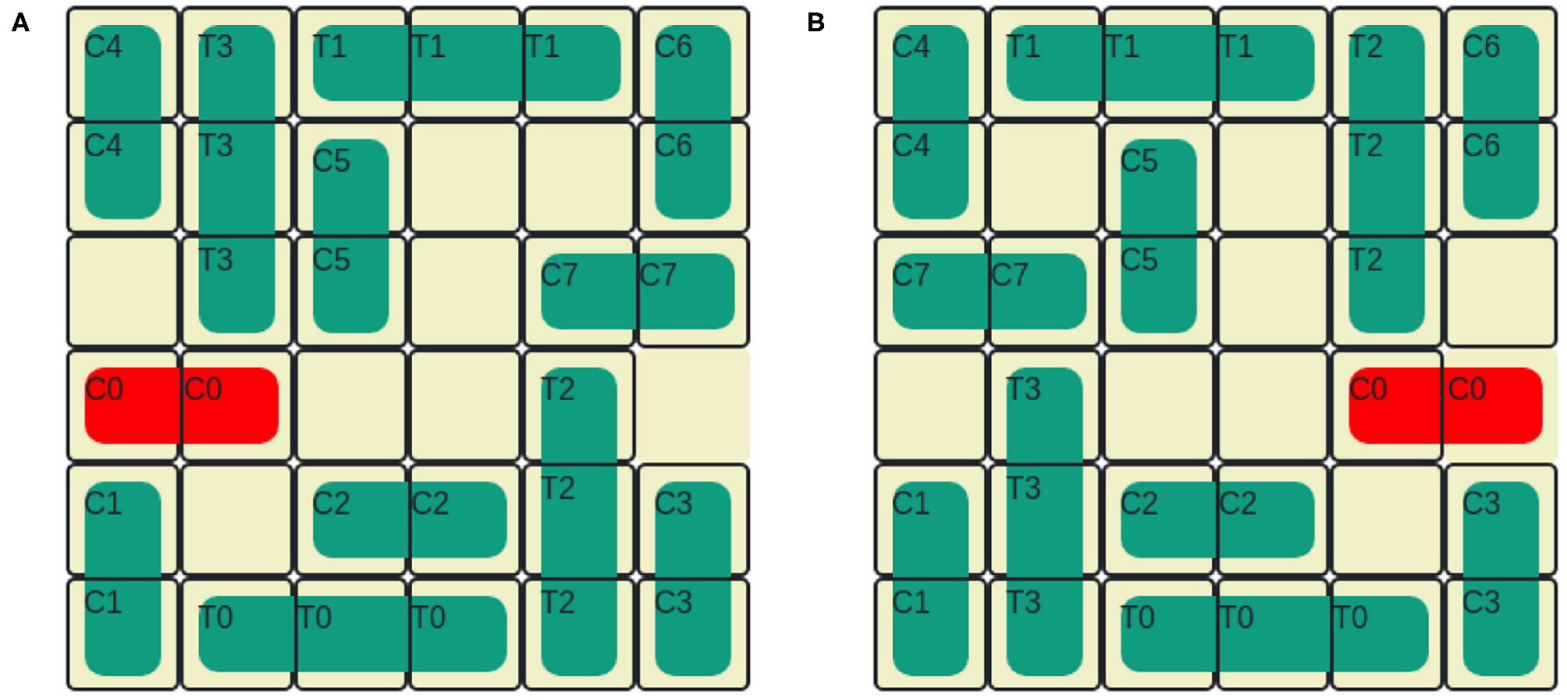
A rush hour instance. **(A)** Initial game stage. **(B)** End game stage.

The Rush Hour puzzle is a game for ages 8 and above (Flake and Baum, 2002). Figure 4A shows an initial puzzle configuration on a 6 × 6 grid, where cars (length 2) and trucks (length 3) are arranged. Vertically aligned vehicles can only move up and down and horizontal vehicles can move left and right. Vehicles can be moved one at a time and into adjacent empty spaces. The solution to the Rush Hour puzzle is a sequence of legal moves that transforms the initial board state shown in Figure 4A to the goal state in Figure 4B. For the puzzle shown in Figure 4, the shortest solution has 21 moves, if the number of moves is considered as the optimizing criteria. If optimized for the number of vehicles moved, the puzzle can be solved optimally by moving only 8 vehicles. It is important to note that one can obtain different “optimal solutions" depending on whether the number of moves is minimized, or if the number of vehicles moved is minimized. Humans tend not to clearly make this distinction when playing the game.

We adopt the formal definition of a Rush Hour instance from Flake and Baum (2002).

**Definition 6**. A Rush Hour instance *is a tuple* 〈w,h,x,y,n,V〉 *such that*:

- (*w, h*) ∈ ℕ^2^ *are the grid dimensions. In the standard version*, *w* = *h* = 6
- (*x, y*), *x* ∈ {1, *w*} and *y* ∈ {1, *h*} *are the coordinates of the exit, which must be on the grid perimeter*.
- *n* ∈ ℕ *the number of non-target vehicles*
- $V=\left\{v_{0},\ldots ,v_{n}\right\}$ *is the set of* *n* + 1 *vehicles comprised of cars* (C) *and trucks* (T). *Note that* |V|=|C|+|T|
  $v_{i}\in C$ *is identified as* {*C*_0_, …, *C*_*l*_}, *where* l=|C|-1 *and* $v_{i}\in T$ *is identified as* {*T*_0_, …, *T*_*m*_}, *where* m=|T|-1.
  *A vehicle is a tuple* *v*_*i*_ = 〈*x*_*i*_, *y*_*i*_, *o*_*i*_, *s*_*i*_〉, *where* $\left(x_{i},y_{i}\right)\in {\mathbb{N}}^{2}$ *are the vehicle coordinates*, *o*_*i*_ ∈ {*N, E, S, W*} *is the vehicle orientation for North, East, South, West*, *s*_*i*_ ∈ {2, 3} *is the vehicle size and* *C*_0_ *is the target vehicle*.

Flake and Baum (2002) also defines the *solution* to a Rush Hour instance as a sequence of *m* moves, where each move consists of a vehicle identifier *i*, a direction that is consistent with the initial orientation of *v*_*i*_, and a distance. Each move, in sequence must be consistent with itself and with the configuration prior to the move. Further, in order to move a distance *d* the configuration must be consistent for all *d*′ such that, 0 ⩽ *d*′ ⩽ *d* (i.e., a vehicle cannot jump over other vehicles on its path).

### 6.2. The Rush Hour Puzzle as a STRIPS Planning Task for Intervention

We study how human users approach solving a cognitively engaging problem as a planning task and evaluate whether we can use domain-specific features to predict intervention. To collect realistic data, it is critical that the human users were willing to participate in the task. The Rush Hour puzzle addresses this requirement well, as evidenced by the feedback about the task we received from the demographic survey. See Section 11.2 in the Appendix for details. Although the Rush Hour puzzle is a game, the environment is rich enough to simulate undesirable consequences and also offer the users a task that is challenging enough.

We translate the Rush Hour puzzle in Definition 6 into a grounded STRIPS planning task *P* = 〈*F, A, s*_0_, *G*〉 as follows:

- *F* = {$\left\{\forall C_{i}\in C,(\mathrm{car}\;?C_{i})\},\;\{\forall T_{i}\in T,(\mathrm{truck}\;?T_{i})\right\}$ - for the vehicles,
  {$\forall v_{i}\in C\cup T$ and *l*_*i*_ ∈ {(*x, y*)|*x* ∈ [1..*w*], *y* ∈ [1..*h*]}, (at ?*v*_*i*_ ?*l*_*i*_)} - for the vehicle positions,
  {$\forall v_{i}\in C\cup T$ and *d*_*i*_ ∈ {*NS, SN, EW, WE*}, (face ?*v*_*i*_ ?*d*_*i*_)} - for direction of vehicles (North to South (down), South to North (up), East to West (left), West to East (right), respectively),
  {∀*l*_*i*_ ∈ {(*x, y*)|*x* ∈ [1..*w*], *y* ∈ [1..*h*]}, (free ?*l*_*i*_)} - for open positions,
  {∀*l*_*i*_, *l*_*j*_ ∈ {(*x, y*)|*x* ∈ [1..*w*], *y* ∈ [1..*h*]} and *d*_*i*_ ∈ {*NS, SN, EW, WE*}, (next ?*d*_*i*_ ?*l*_*i*_ ?*l*_*j*_)} - for direction of the adjacent locations) }
- *A* = {move-car = 〈pre(move-car), add(move-car), del(move-car)〉 ⊆ *F*,
  move-truck = 〈pre(move-truck), add(move-truck), del(move-truck)〉 ⊆ *F*}
- *s*_0_ ⊆ *F*
- *G* = {(at *C*_0_ *l*_*i*_)(at *C*_0_ *l*_*j*_)}, where *l*_*i*_ = (*w*, 3) and *l*_*j*_ = (*w*−1, 3)

In order to configure the Rush Hour STRIPS planning task for intervention, we introduce an undesirable state, u by designating one vehicle as *forbidden*. The post-conditions of any action that moves the forbidden vehicle satisfies u. The puzzle can be solved without moving the forbidden vehicle. Therefore, moving the forbidden vehicle is also an unnecessary action, indicating that the user is exploring an unhelpful region in the state space. Here, intervention is required to guide the user toward exploring more helpful regions in the state space. In Figure 4A, the forbidden vehicle is *C*_2_. If the user moves *C*_2_ to the left, then the board state satisfies u. The user's goal d = *G*. The vehicle movement constraint introduced by the presence of the forbidden vehicle adds an extra level of difficulty to the user's planning task.

The Rush Hour problem is also unique in that the observer is more focused on states than actions. Recall that a history *H* = (*o*_1_[*s*_1_], *o*_2_[*s*_2_], …, *o*_*i*−1_[*s*_*H*_]) is a sequence of previously observed actions, which started from *s*_0_ with the implied resulting states in brackets. For Rush Hour, the observer relies on those implied states instead of just the actions. For simplicity in notation, we present intervention in terms of states, although it is easy to map between actions and states because of the deterministic state transition system of the planning model.

### 6.3. Domain-Specific Feature Set

For the observer to learn *intervene*_*i*_, we develop a set of domain-specific features for the Rush Hour problem. We want the feature set to capture whether the user is advancing toward d by making helpful moves, or whether the user currently exploring a risky part in the state space and getting closer to u. We hypothesize that the behavior patterns extracted from *H* as features have a correlation to the event of the user moving the forbidden vehicle.

#### 6.3.1. Features Based on State

The features based on state analyze the properties of the sequence of state transitions in *H* from *s*_0_ to *s*_*H*_ ([*s*_0_], [*s*_1_], [*s*_2_], …, [*s*_*H*_]). Specifically, we look at the *mobility* of the objects: target vehicle (*C*_0_), the forbidden vehicle and the vehicles adjacent to the target and the forbidden vehicles. We use the state features associated with the target vehicle to measure how close the user is to d. The state features associated with the forbidden car evaluate how close the user is to triggering u.

We manually examined the solutions produced in the human subject experiment (described later) to identify common movement patterns. Our analysis revealed that if the user was moving vehicles adjacent to the forbidden vehicle in such a way that the forbidden vehicle was freed, most users ended up moving the forbidden vehicle. Therefore, by monitoring the state changes occurring around the forbidden vehicle, we can estimate whether the user will end up moving the forbidden vehicle or not (i.e., trigger u). Similarly, state changes occurring on the target car's path to the exit, for example, the moves that result in reducing the number of vehicles blocking the target car is considered to be helpful to move the state closer to d.

**Figure 5:**
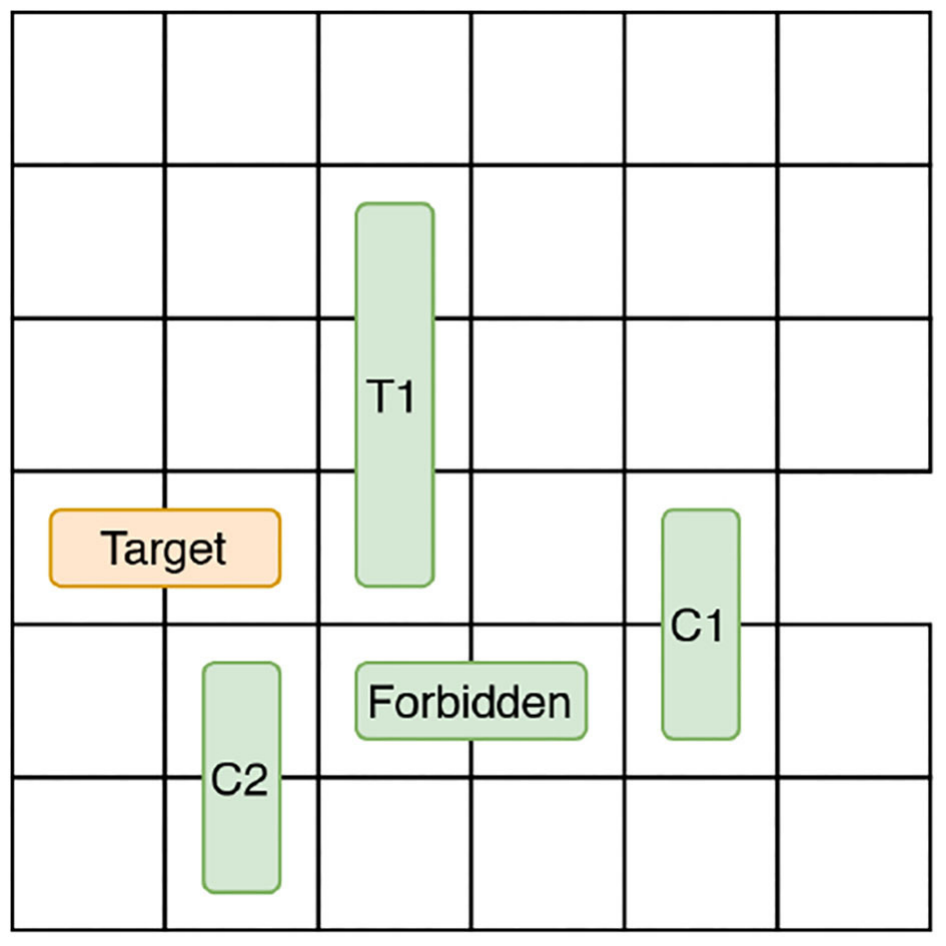
Blocker vehicles.

We refer to the vehicles adjacent to the target and the forbidden vehicles as *blockers* and introduce two additional object types to monitor mobility: *target car blockers* and *forbidden car blockers*. Figure 5 illustrates an example state. The target car's path is blocked by two vehicles *C*_1_ and *T*_1_. Therefore, target car blockers = {*C*_1_, *T*_1_}. We only consider the vehicles that are between the target car and the exit cell as target car blockers because, only those vehicles are preventing the target car from reaching d. The forbidden vehicle's movement is blocked by two vehicles *C*_1_ and *C*_2_. Therefore, forbidden car blockers = {*C*_1_, *C*_2_}. We now describe the features based on state that are used to predict intervention in Human-aware Intervention Problems.

- blocks: number of times a move increased the number of cars blocking the target car's path
- frees: number of times a move freed up empty spaces around the forbidden vehicle
- freebci: number of times the number of empty spaces around the forbidden vehicle blockers increased
- freebcd: number of times the number of empty spaces around the forbidden vehicle blockers decreased
- freegci: number of times the number of empty spaces around the target car blockers increased
- freegcd: number of times the number of empty spaces around the target car blockers decreased
- mgc: mean number of empty spaces around the target car blockers
- mbc: mean number of empty spaces around the forbidden car blockers
- reset: number of times the current move changed the state back to the initial puzzle configuration

#### 6.3.2. Features Based on User Actions

The features based on user state analyze the properties of the sequence of actions from *o*_1_ to *o*_*i*−1_ in *H* (*o*_1_, *o*_2_, …, *o*_*i*−1_) We follow the same manual analysis of solutions produced in the human subject experiment to identify common movement patterns. We found that the users who produce unsafe solutions often made unhelpful moves such as moving the same vehicle back and forth many times in quick succession, causing their solution to be longer compared to a safe solution. We statistically verified whether the relationship between the solution length and the number of forbidden vehicle moves is significant for the human subject data using Spearman's Rank Correlation Coefficient. The test showed that the relationship is significant (*p* < 0.05). See Section 11.3 in the Appendix for a summary of raw data.

Similarly, we observed that comparing the number of moves of the user's solution to an optimal solution produced by an automated planner is helpful in identifying whether the user is moving away from d or making progress. In order to verify this observation, we use the HSP planner (Bonet and Geffner, 2001) to find cost optimal solutions for the Rush Hour planning tasks (see Section 6.2) used in the human subject experiment. We statistically verified that the relationship between the length difference between the user's solution and the optimal solution found by an automated planner, and the number of forbidden vehicle moves is significant using Spearman's Rank Correlation Coefficient (*p* < 0.05).

Thus, we conclude that features derived from the length and number of backtracking moves in *H* can be used to predict when the user is getting close to u. We introduce an unhelpful move called the *h-step backtrack*, which is a move that takes the state back to a previously seen state by *h* number of steps (i.e., an undo operation). When deriving the feature to capture backtracking moves, we only consider *h* = 1, which asks the question did the observation *o*_*i*−1_ undo the effect of the observation *o*_*i*−2_?

We now describe the features based on actions that are used to predict intervention in Human-aware Intervention Problems.

- len: number of moves in *H*
- len-opt: difference of the number of moves in *H* and the number of moves in the safe optimal solution produced by an automated planner for the same planning task.
- backtracks: number of 1-step backtrack actions in *H*
- first: number of moves until the forbidden vehicle was moved for the first time in *H*
- prop: number of moves until the forbidden vehicle was moved for the first time in *H* divided by the number of moves in the safe, optimal solution produced by an automated planner for the same planning task.
- moved: number of vehicles moved in *H*.

## 7. Evaluating Human-Aware Intervention

To evaluate the efficacy of the features based on state and features based on actions in predicting intervention for Human-aware Intervention Problems, we use actual observation traces collected from a human subject study, where human users solve Rush Hour planning tasks on a Web simulator. We generate learned models that predict intervention while offering different levels of freedom to the user. We consider three levels of freedom (*k* = {1, 2, 3}) for the evaluation. A model for the lowest level of freedom (*k* = 1), predicts intervention one move before the undesirable state. This configuration offers no time for the user to recover from the undesirable state. A model for the next level of freedom (*k* = 2), intervenes the user two moves before the undesirable state is satisfied and offers the user some time to take corrective action. A model for the highest level of freedom (*k* = 3), intervenes the user three moves before the undesirable state. We begin with the experiment protocol and briefly describe the findings. Next, we discuss the learning methods used to predict intervention. Finally, we discuss the accuracy of prediction compared to the Plan Recognition as Planning algorithm proposed by Ramırez and Geffner (2010).

### 7.1. Rush Hour Experiment Protocol

We recruited subjects from a university student population. The sample comprised of college students in Computer Science, Psychology, Agriculture and Business majors. One hundred and thirty six participants completed the study. The participants were not compensated for their time. After obtaining informed consent, the participants were directed to the Web URL (https://aiplanning.cs.colostate.edu:9080/), which hosted the Rush Hour simulator software. Each participant was assigned to solve one randomly selected Rush Hour puzzle. We did not place any time restriction for the puzzle solving task. Participants also had the option to use an online tutorial (available on the Web simulator application) on how to play the Rush Hour puzzle. Each puzzle contained one forbidden vehicle. Once the puzzle solving task was completed, the participants were asked to complete a short demographic survey on their general puzzle solving habits. One hundred and seventeen of the 136 participants also completed the demographics survey.

**Figure 6:**
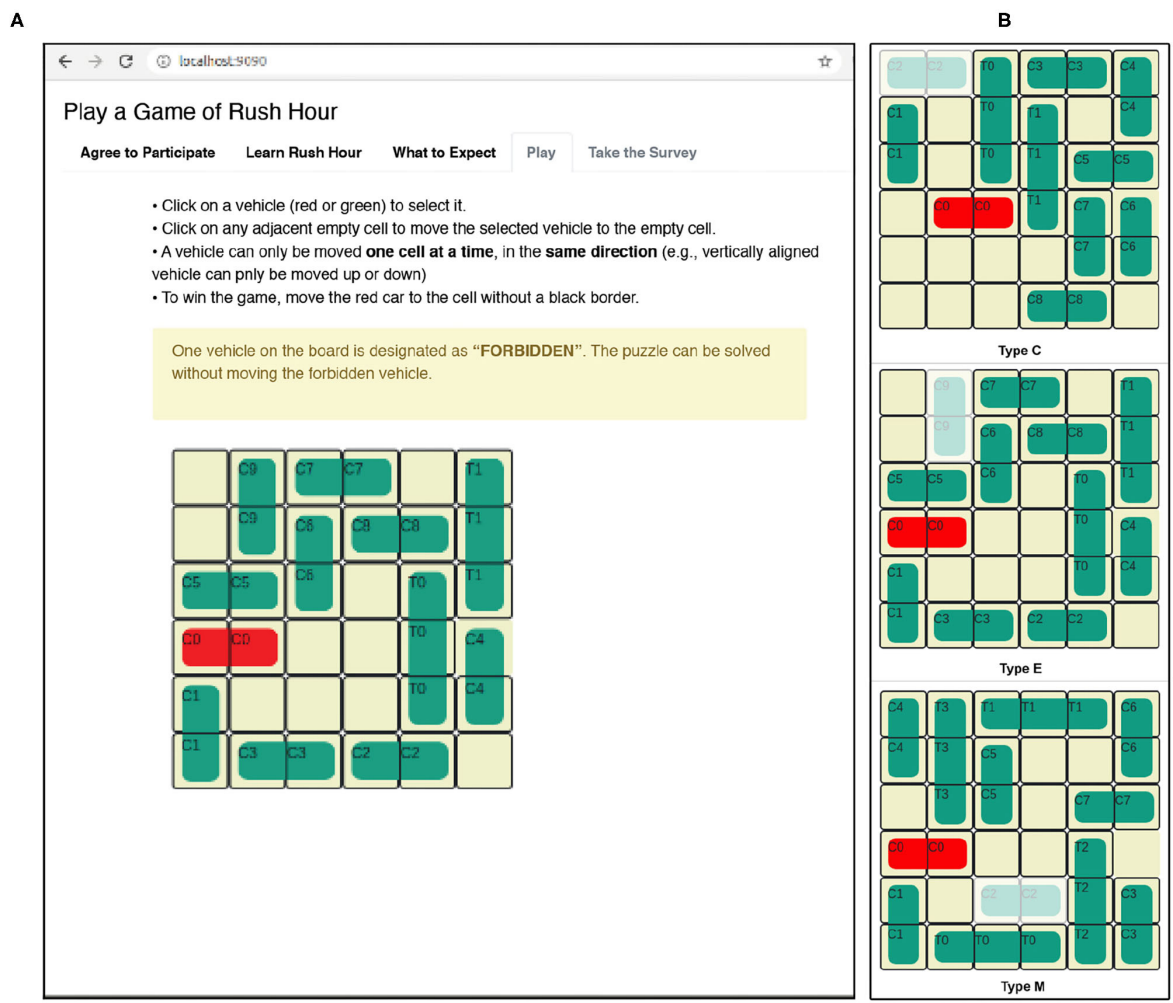
**(A)** Rush Hour planning task Web interface. The forbidden vehicle for this configuration is *C*_9_. **(B)** Rush Hour puzzle configuration types with forbidden vehicles highlighted.

When choosing Rush Hour puzzle instances for the human subject study, we want to carefully balance the puzzle's difficulty for a human user. Especially, considering the PSPACE-completeness of the (generalized) puzzle, we need the puzzles to be solvable by human users in a reasonable time. We used a pilot study to determine the puzzle difficulty. See Section 11.1 in the Appendix. We ensured that the experiment protocol fully adhered to the Rush Hour planning task definitions (Section 6.2). The goal of the Rush Hour planning task (d) is clearly communicated to the user. To instill the importance of avoiding the forbidden vehicle in the user's mind, we provided an information message (yellow information bar in Figure 6) to inform about the presence of a forbidden vehicle without specifying the vehicle identifier. The users were also informed that the puzzle can be solved without moving the forbidden vehicle. If the user moved the forbidden vehicle, no visual cues (error messages, blocks) were given. Therefore, the specific undesirable state (u) remained hidden to the user, but they were made aware of its presence. We informed the human users that there is a forbidden vehicle and they must try to solve the puzzle without moving it to prime them toward thinking more deeply about the puzzle and *raise the awareness of raise the aware of the undesirable state*. This technique allowed us to increase the cognitive load on the human user because it conditioned the human users to study the puzzle to guess/recognize the forbidden vehicle. Further it primed the users to make thoughtful moves instead of making random moves and accidentally finding the solution.

To simulate discrete actions, the vehicles on the board could only be moved one cell at a time. The user could click on the object to select it and move it by clicking on an empty adjacent cell. Invalid moves (vehicle dragging and jumps) were blocked and the user was notified via an alert message. We recorded the user's solution to the STRIPS planning task as a sequence of actions in a text file.

We used ten Rush Hour planning tasks for the experiment. For analysis purposes (see Appendix), we separated the ten puzzles into three groups by the position of the forbidden vehicle. As shown in Figure 6B, type **C** has the forbidden vehicle in the **c**orner of the board. Type **E** has the forbidden vehicle on an **e**dge. Type **M** has the forbidden vehicle in the **m**iddle. The experiment used four puzzles of type C, five puzzles of type E and one puzzle of type M.

### 7.2. Length Distributions of Human Users' Solutions

We now compare the cost of the solutions produced by human users by dividing the solutions into two types: safe and unsafe. In this analysis, we assumed each move is unit cost, therefore the cost of the solution is equal to the number of moves. We refer to solutions that did not move the forbidden vehicle as safe and solutions that moved the forbidden vehicle as unsafe. Sixty six users from the total 136 solutions that involved moving the forbidden vehicle (49%). From those who moved the forbidden vehicle, 54 users moved the vehicle more than once (82%). Table 9 describes the summary statistics for the safe and unsafe solutions.

**Table 9:** Frequency, minimum, maximum, mean, and standard deviation (SD) of the number of moves in human user solutions for the Rush Hour planning tasks P1 through P10.

**PID**	**Safe**					**Unsafe**				
	**Freq**	**Min**	**Max**	**Mean**	**SD**	**Freq**	**Min**	**Max**	**Mean**	**SD**
P1 (E)	18	24	106	43.9	20.5	-	-	-	-	-
P2 (C)	3	44	158	99.3	57.1	8	78	378	190.3	120
P3 (E)	-	-	-	-	-	12	25	50	35.5	8.3
P4 (C)	9	23	46	30	7.1	7	25	124	67.4	33.9
P5 (E)	4	23	32	26.5	3.9	7	14	82	32.0	23.3
P6 (C)	14	22	55	29	10.3	-	-	-	-	-
P7 (M)	2	29	37	33	5.7	9	43	132	80.9	38.2
P8 (C)	18	9	12	9.3	0.8	-	-	-	-	-
P9 (E)	2	21	27	24	4.2	14	29	169.0	66.3	39
P10 (E)	-	-	-	-	-	9	44	158	81.2	39.2

*The letter within parenthesis next to each puzzle id indicate the puzzle type*.

We see that the planning task P1 did not produce any unsafe solutions. The reason for this observation is that when examining the structure of P1 it can be seen that moving the forbidden vehicle makes the planning task unsolvable. Second, the planning task P8 did not produce any unsafe solutions. Furthermore, P8 and P6 planning tasks in type C did not produce any unsafe solutions. Human users found it difficult to avoid the forbidden car for type E planning tasks, where the forbidden car was positioned along the edge of the board. For type E planning tasks (P3, P5, P9, P10), there are more unsafe solutions than safe solutions and also the mean solution length for unsafe solutions is larger compared to the safe solutions mean. We only had 1 planning task for type M (P7), where the forbidden vehicle was placed in the middle of the board. The users who attempted P7 found it difficult to solve the planning task without moving the forbidden vehicle.

**Figure 7:**
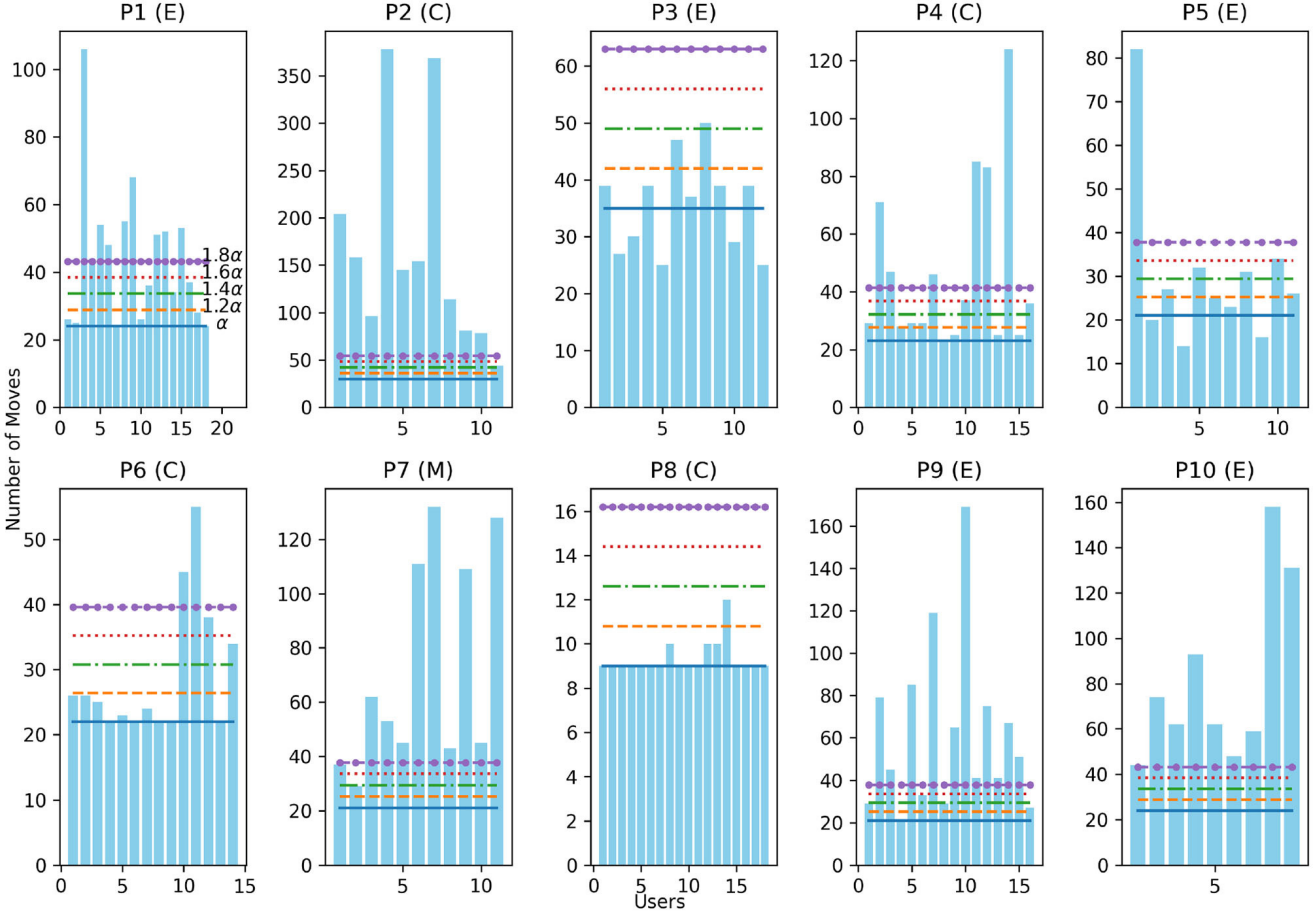
Number of moves in the users' solution compared to the optimal number of moves α, 1.2α, 1.4α, 1.6α, 1.8α for puzzles P1 through P10.

Figure 7 illustrates how the number of moves in users' solutions compare to a set of threshold values derived from the optimal solution for each Rush Hour planning task. We define the threshold set θ as: given the optimal number of moves α for a puzzle, θ = {α, 1.2α, 1.4α, 1.6α, 1.8α}. The letter in parenthesis indicates the puzzle group (see Figure 6B for the three puzzle types) of each puzzle.

It can be seen that human solvers' solutions to P8 were very close to the optimal solution in the number of moves. Human solvers' found it very difficult to find a solution closer to the optimal number of moves for P2. Users who attempted P3 and P5 found solutions shorter than the safe, optimal. Shorter solutions for these two puzzles all required the user to move the forbidden vehicles. This observation allows us to draw a conclusion that the recovery process of the Human-aware Intervention needs to aim at reducing the remaining number of moves the user has to execute to help them avoid the forbidden vehicle.

### 7.3. The Learning Methods

Our solution to the Human-aware Intervention Problem uses machine learning to predict whether u will be reached in *k* moves, given *H*, where *k* = {1, 2, 3}. To produce the learned models, we first partition the 136 human user solution from the experiment into training (70%) and test (30%) sets. To produce the *H* for a user, the user's solution is pre-processed to only include the moves until one step, two steps and three steps before the forbidden vehicle was moved for the first time. For example, in a solution *O* = {*o*_1_, …, *o*_*i*_}, if a user moved the forbidden vehicle in step *i*, we generate three observation traces *O*_1_ = {*o*_1_, …, *o*_*i*−1_}, *O*_2_ = {*o*_1_, …, *o*_*i*−2_} and *O*_3_ = {*o*_1_, …, *o*_*i*−3_} corresponding to that user. Observation traces of type *O*_1_ were used to train the model for *k* = 1, observation traces of type *O*_2_ were used to train the model for *k* = 2 and so on. Given the sequence of actions in the user's solution, the corresponding state after each move required for *H* is derived using the STRIPS planning model for the corresponding Rush Hour puzzle. We use the features based on state and features based on actions together to train five classifiers with 10-fold cross validation for each value of *k*. We explore a number of classifiers: the decision tree, K-nearest neighbor, Logistic Regression and Naive Bayes. The classifiers are used in the supervised learning mode. We summarize the parameters used in each learning method below:

- *Decision Tree:* We use the J48 classifier available on the WEKA platform (Hall et al., 2009). This classifier implements the C4.5 algorithm (Quinlan, 1993). The decision tree classifier is tuned to user pruning confidence = 0.25 and the minimum number of instances per leaf = 2.
- *k Nearest Neighbor (KNN):* We use this classifier with a Euclidean distance metric, considering the value *k* = 1
- *Logistic Regression:* This classifier is tuned for ridge parameter = 1.0*E* − 8.
- *Naive Bayes:* This classifier is tuned with the supervised discretization = True.

#### 7.3.1. Human-Aware Intervention Accuracy

We used the learned models on the test data set to predict whether the observer should intervene given *H*. In order to evaluate the classifier accuracy, we first define true-positives, true-negatives, false-positives, false-negatives for the Human-aware Intervention Problem. A true-positive is when the classifier correctly predicts that u will be reached in *k* moves given *H*. A true-negative is when the classifier correctly predicts that the u will not be reached in *k* moves. A false-positive is when where the classifier incorrectly identifies that the u will be reached in *k* moves. A false-negative is when the classifier identifies that the u will not be reached in *k* moves, but in fact it does.

Table 10 summarizes the precision, recall and F-score for predicting intervention for *k* = {1, 2, 3}. It can be seen that the Logistic Regression classifier performs the best with high precision/recall compared to other classifiers when predicting intervention for {*k* = 1, 2} When *k* = 3, the Logistic Regression classifier predicts intervention with high recall but slightly lower precision. The precision of the decision tree classifier improves for higher values of *k*. However, the recall and F-score drop when *k* = 3. The Naive Bayes classifier reported the lowest precision compared to all other classifiers for any value of *k*.

**Table 10:** Precision, Recall, and F-scores for the prediction accuracy of the Human-aware Intervention learned models.

**Classifier**	***k*** = 1			***k*** = 2			***k*** = 3		
	**Precision**	**Recall**	**F-score**	**Precision**	**Recall**	**F-score**	**Precision**	**Recall**	**F-score**
Decision tree	0.70	0.90	0.89	0.80	0.95	0.87	0.89	0.81	0.85
KNN	0.89	0.76	0.82	0.86	0.86	0.86	**0.95**	**0.90**	**0.93**
Logistic regression	**0.91**	**0.95**	**0.93**	**0.87**	**1**	**0.93**	**0.91**	**0.95**	**0.93**
Naive bayes	0.73	0.90	0.81	0.74	0.86	0.83	0.68	0.90	0.78

*Classifiers that reported the highest precision, recall and F-score are in bold. When k = 3, both the KNN and logistic regression classifiers have the same F-score. However, KNN has the best precision. The best recall value is reported for the logistic regression classifier. Therefore, both are highlighted*.

#### 7.3.2. Human-Aware Intervention as a Plan Recognition Problem

Recall that in Section 2, we showed that it is possible to frame the Intervention Problem as a Plan Recognition task, where the observer models the u and d as the set of likely goals of the user. Then, we can implement the observer in the Rush Hour puzzle solving task as a probabilistic Plan Recognition agent that solves the Plan Recognition Problem $T=\langle D_{\text{user}},G,H,Prob\rangle$, where *D*_user_ is the planning domain, the set of likely goals G={u,d}, *H* is the observation sequence and *Prob* is a probability distribution over G. By compiling the observations away into the domain theory, Ramirez and Geffner showed that it is possible to use an automated planner to find plans that are compatible with the observations and use these observation compatible plans to determine what the most likely goal and plan (Ramırez and Geffner, 2010) is for the user. They evaluated the approach on benchmark planning domains.

If the observer can recognize u as the most likely goal given the observations, then the observer must intervene at that point. We adapt the Plan Recognition as Planning (PRP) algorithm proposed by Ramırez and Geffner (2010), to evaluate how the observer executing PRP recognizes the most likely goal given the observation traces *O*_1_ = {*o*_1_, …, *o*_*i*−1_}, *O*_2_ = {*o*_1_, …, *o*_*i*−2_}, and *O*_3_ = {*o*_1_, …, *o*_*i*−3_}, corresponding to *k* = {1, 2, 3}. We use the same test set from the classifier evaluation to evaluate the PRP algorithm in predicting intervention. This experiment also allows us to evaluate the PRP algorithm on plans generated by human users.

In order to evaluate the PRP accuracy, we first define true-positives, true-negatives, false-positives, false-negatives for the observer implementing the PRP algorithm. A true-positive is when the observer correctly selects u as the most likely goal given the observation traces *O*_1_, *O*_2_ and *O*_3_ for {*k* = 1, 2, 3}, respectively. A true-negative is when the observer correctly does not select u as the most likely goal given the observation traces. A false-positive is when the observer incorrectly identifies u as the most likely goal given the observations. A false-negative is when the observer incorrectly does not select u as the most likely goal but in fact it is.

Table 11 summarizes the precision, recall and F-score for deciding to intervene correctly for *k* = {1, 2, 3} using PRP. PRP requires goal priors as an input to the algorithm. We used the LAMA planner (Richter and Westphal, 2010) to generate satisficing plans that are compatible with the observations for PRP. Our assumption of the users' plans being satisficing is justified by the findings reported in Section 7.2. It shows that the majority of the users did not find optimal length solutions. In order to analyze the algorithm performance on different goal priors, we set three levels: (1) uniform goal priors, where u and d are equally likely (i.e., *P*(u) = *P*(d)), (2) u is more likely and (3) d is more likely. We decided on these prior probability distributions based on the forbidden vehicle's position on initial game configurations used in the experiment. We set a higher value for u to indicate the situation where the forbidden vehicle is not blocked by other vehicles. As a result, the user will likely move it. We set d as high if the forbidden vehicle is blocked in the initial configuration, to indicate that the user will be less likely to move it. The uniform probability is the default.

**Table 11:** Precision, Recall, and F-scores for solving the Human-aware Intervention problem as a Plan Recognition problem with the Plan Recognition as Planning algorithm (Ramırez and Geffner, 2010).

**Goal priors**	***k*** = 1			***k*** = 2			***k*** = 3		
	**Precision**	**Recall**	**F-score**	**Precision**	**Recall**	**F-score**	**Precision**	**Recall**	**F-score**
Uniform	0.67	0.56	0.61	0.67	0.56	0.61	0.56	0.67	0.61
*P*(u) = 2×*P*(d)	0.69	0.61	0.65	0.69	0.61	0.65	0.69	0.61	0.65
*P*(d) = 2×*P*(u)	0.67	0.56	0.61	0.67	0.56	0.61	0.67	0.56	0.67

It can be seen that PRP accuracy in predicting when the u will be reached is lower compared to the learned models for all levels of *k*. The accuracy does not change with values of *k*. This observation is consistent with the accuracy of the logistic regression model (i.e., the best performing learned model). When the goal priors are biased in favor of the undesirable state, the prediction accuracy slightly improves. However, giving a higher prior to u contradicts with our intervention models' assumptions that the user wants to avoid the undesirable state, which implies that *P*(u) must be low.

## 8. Related Work

We discuss prior research related to the The Intervention Problem beginning with plan/goal recognition. This is because in order to intervene, the observer must first recognize what the actor is trying to accomplish in the domain. In the two intervention models we have proposed: Unsafe Suffix Analysis Intervention and Human-aware Intervention, the observer has limited interaction with the actor. For example, upon sensing an action and determining that it requires intervention the observer simply executes the accept-observation action to admit the observed action into *H*. In real-life situations, helpful intervention requires more observer engagement, i.e., an active observer to help the actor recover from intervention. Therefore, we discuss existing work on designing active observers having both the recognition and interaction capabilities. Next, we discuss related work on dealing with misconceptions held by the actor about the planning domain and AI safety in general. Because we specially focus on developing intervention models for human users, we discuss related work on using machine learning algorithm to classify human user behavior and how intervention is leveraged to provide intelligent help to human users.

### 8.1. Plan Recognition

The Plan Recognition Problem is to “*take as input a sequence of actions performed by an actor and to infer the goal pursued by the actor and also to organize the action sequence in terms of a plan structure*” (Schmidt et al., 1978). Early solutions to the Plan Recognition problem require that the part of a plan given as input to the recognizer be matched to a *plan library*. Generalized Plan Recognition (Kautz and Allen, 1986), identifies a minimal set of top-level actions sufficient to explain the set of observed actions. The plans are modeled in a graph, where the nodes are actions and there are two types of edges: action specialization and decomposition. The Plan Recognition task then becomes the minimum vertex cover problem of the graph. Geib and Goldman (2009) represent the plan library for a cyber security domain as partially ordered AND/OR trees. In order to recognize the actor's plan, the recognizer needs to derive a probability distribution over a set of likely explanation plans π given observations *O*, *P*(π|*O*). To extract the likely explanation plans, the authors define a grammar to parse the AND/OR trees and generatively build the explanations by starting off with a “guess” and refining it as more observations arrive. The actor is hostile to the recognition task and will hide some actions, causing the recognizer to deal with partial observability when computing *P*(π|*O*).

One concern with using plan libraries for recognition is the noise in the input observations. The case-based Plan Recognition approach (Vattam and Aha, 2015) relaxes the error-free requirement of the observations and introduces a recognition algorithm that can handle missing and misclassified observations. The solution assumes that there exists a plan library consisting of a set of cases stored using a labeled, directed graph called *action sequence graphs*, which is an encoding of an action-state sequence that preserves the order of the actions and states.

Ramırez and Geffner (2010) proposed a recognition solution that does not rely on defining a plan library. By compiling the observations away into a planning language called PDDL, their approach exploits automated planners to find plans that are compatible with the observations. Ramirez and Geffners' solution recognizes the actor's goals (and plans) by accounting for the cost differences of two types of plans for each candidate goal: (1) plans that reach the goal while going through the observations and, (2) plans that reach the goal without going through the observations. Ramirez and Geffner characterize the likelihood *P*(*O*|*g*) as a Boltzmann distribution $P\left(O|g\right)=\frac{e^{-\beta \Delta_{g}}}{1+e^{-\beta \Delta_{g}}}$ where β is a positive constant. Sohrabi et al. (2016a) propose two extensions to the Ramirez and Geffener's Plan Recognition work. First, the recognition system can now handle noisy or missing observations. Their new approach to “Compiling Observations Away” modifies the planning domain to include action costs; specifically penalties for noisy/missing observations. Second, recognition is defined for observations over state variables.

In plan recognition, the recognizer reasons about the likely goals from a set of goal hypotheses given the actor's behavior. The recognizer's task may fail if he cannot accurately disambiguate between possible goal hypotheses. Mirsky et al. (2018) propose sequential plan recognition, where the user is sequentially queried in real-time to verify whether the observed partial plan is correct. The actor's answers are used to prune the possible hypotheses, while accounting for the incomplete plans that could match with the observations after several other observations happen in the future. In order to optimize the querying process, the recognizer considers only the queries that maximize the information-gain and the likelihood of the resulting hypotheses. This solution assumes that a plan library is available. Their implementation of the plan library uses trees to represent the possible plans for goal hypotheses. Sequential plan recognition is not really suited for the intervention scenarios we discuss in this work, because we assume that the undesirable state that must be avoided is hidden to the user.

Online goal recognition (Vered and Kaminka, 2017) extends the recognition problem to continuous domains where the recognition problem must be solved for every new observation when they are revealed. Formally, we seek to determine the probability of a goal *g* given observations *O*, *P*(*g*|*O*) for each goal g∈G. The recognized goal is the one that has the highest posterior probability. Instead of taking the cost difference (Ramirez and Geffners' approach) they define a ratio $score\left(g\right)=\frac{cost\left(i_{g}\right)}{cost\left(m_{g}\right)}$, where *i*_*g*_ is the *optimal* plan to achieve *g* and *m*_*g*_ is the *optimal* plan that achieves *g* and includes all the observations. When the optimal plan that has all the observations is the same cost as the optimal the score approaches 1. Then *P*(*g*|*O*) = η*score*(*g*), where η is the normalizing constant. Optimal plan *i*_*g*_ can be computed using a planner. To compute *m*_*g*_, they exploit the fact that each observation is a trajectory or point in the continuous space and each likely plan is also a trajectory in the same space. Therefore, *m*_*g*_ = *prefix* + *suffix*, where *prefix* is built by concatenating all observations in *O* into a single trajectory, and the *suffix* is generated by calling a planner from the last observed point to goal *g*. Follow up work further reduces the computational cost of online goal recognition by introducing *landmarks* to prune the likely goals (Vered et al., 2018). Landmarks are facts that must be true at some point in all valid plans that achieve a goal from an initial state (Hoffmann et al., 2004). Goal recognition is performed by using landmarks to compute the completion ratio of the likely goals as a proxy for estimating *P*(*g*|*O*).

### 8.2. Goal/Plan Recognition With an Active Observer

While the goal/plan recognition works discussed in the previous section assume a passive observer, a growing body of work has also looked into recognition problems with active observers. Only recognizing when intervention is needed (as a passive observer) solves only a part of the problem. In cases where intervention is used for an artificial agent, active observers can force the agent to alter its current plan. When intervention happens during a cognitively engaging task, as in the Rush Hour puzzle, a human user would naturally like to know what to do next. An active observer who can take action or give instructions to the human user, not only will be able to assist the user complete the task safely but also will improve the human user's interaction with the AI system.

Bisson et al. (2011) propose a plan library based plan recognition technique to provoke the observed agent so that it becomes easier to disambiguate between pending goal hypotheses. The observer modifies the fluents associated with a *provokable* action, which forces the observed agent to react on the modification. The provokable event is selected heuristically such that it reduces the uncertainty among the observed agent's likely goals. In another approach that aims to expedite the goal recognition, Shvo and McIlraith (2020) use landmarks to eliminate hypothesized goals. They define the Active Goal Recognition problem for an observer agent who can execute sensing and world-altering actions. The observer executes a *contingent plan* containing the sensing and world-altering actions to confirm/refute the landmarks of the planning problems for each goal hypothesis. Goals hypotheses whose landmarks (for the corresponding planning problem) are refuted by the execution of the contingent plan are removed from the set of likely goals. Although the initial problem definition assumes that the observer's contingent plan is non-intervening and is primarily used to reduce the goal hypotheses, Shvo and McIlraith (2020) also propose an extension where the observer can actively impede or aid the actor. For example, the authors suggest adopting the Counter-planning Algorithm proposed by Pozanco et al. (2018) to generate a plan for the observer to impede the actor, after the actor's goals are identified through Active Goal Recognition. Pozanco's Counter-planning Algorithm is designed for a domain where two adversarial agents (seeking and preventing) pursue different goals. In the context of the Active Goal Recognition problem, the seeking agent is the actor while the preventing agent is the observer. Counter-planning requires that the observer quickly identify the seeking agent's goal. They use the Ramirez and Geffener's probabilistic goal recognition algorithm to perform goal recognition. Then the preventing agent actively intervenes the seeking agent by identifying the earliest landmark for the seeking agent's planning problem (for the recognized goal) that needs to be blocked (i.e., counter-planning landmark). The recognizer uses automated planning to generate a plan to achieve the counter-planning landmark (e.g., negating the landmark), thus blocking the seeking agent's goal achievement. The aforementioned works in Active Goal Recognition assume full observability over the actor. Amato and Baisero (2019) relax this constraint and propose Active Goal Recognition with partial observability over the actor and model the planning problem as a partially observable Markov decision process (POMDP) (Kaelbling et al., 1998). Similar to the previously discussed Active Goal Recognition problems, the observer agent is trying to reach it's own goal as well as correctly predict the chosen goal of the actor. Therefore, they define the Active Goal Recognition problem for the observer by augmenting the observer's action space with the actor's actions, the observer's own actions and the decision actions on the actor's goals. The state space is defined as the Cartesian product of the observer's states, actor's states and actor's goals. The goals for the recognition problem are augmented with the observer's own goals. and the prediction of the actor's goals. A solution to this planning problem starts at the the initial states of the observer and the actor and chooses actions to the augmented goal while minimizing the cost (or maximizing a reward). A POMDP is defined to solve the augmented planning problem (i.e., Active Goal Recognition problem).

The goal recognition algorithms discussed above mainly focus on pruning the pending goal hypotheses to allow the observer quickly disambiguate between goals. To accomplish this objective, Shvo et al. use sensing and world-altering actions to confirm/refute the landmarks. Counter-planning also uses landmarks. Bisson et al. use heuristics. Other solutions for goal recognition take a decision theoretic approach where the observer attempts to find plans to achieve own goals while predicting the user's goal optimizing over some reward function. Our intervention models differ from these solutions in the intervention recognition task because we do not prune the goal hypotheses. Instead, we emphasize on accurately recognizing whether an actor's revealed plan is unhelpful (and must be interrupted) where the plans leading to the goal hypotheses share common prefixes, making the disambiguation difficult. We use machine learning to learn the differences between the helpful and unhelpful plan suffixes and use that information to decide when to intervene. We rely on the same plan properties as existing recognition algorithms to learn the differences between plan suffixes: plan cost and landmarks. In addition, we have shown that the plan distance metrics can also be used to differentiate between helpful and unhelpful plan suffixes.

The next step in our work is to extend the Human-aware Intervention model so that the observer can actively help the human user modify his plan following the recognition phase. The works we discussed in this section have already addressed this requirement in agent environments, where the observer also executes actions to support the goal recognition process. Pozanco et al. take a step further to show that following recognition, the observer can impede the actor using planning. Freedman and Zilberstein (2017) discuss a method that allows the observer to interact with the actor while the actor's plan is in progress with fewer observations available. Our experiments validate their argument that plan/goal recognition by itself is more useful as a post-processing step when the final actions are observed, which will be too late for the Intervention problems we discuss in this work. Our solution addresses this limitation, allowing the observer to recognize “before it's too late” that the undesirable state is developing. We use machine learning to perform the recognition task. In contrast, Freedman and Zilberstein (2017) propose a domain modification technique (similar to Ramirez and Geffner's) to formulate a planning problem that determines a relevant interactive response from the current state. Plans that agree (and do not agree) with the observations can now be found using an off-the-shelf planner on the modified domain. The actor's goal is recognized by comparing the costs of these plan sets. Following the recognition phase, they also define assistive and adversarial responsive actions the observer can execute during the interaction phase. Assistive responsive action generation is more related to our Intervention problem because our observer's goal is to help the actor avoid the undesirable state. The authors define an assistive interaction planning problem to generate a plan from the current state for the observer. This *assistive* plan uses the combined fluents of the actor and the observer, the Cartesian product of the actor's and the observer's actions (including no-op actions) and a modified goal condition for the observer. The assistive action generation through planning proposed by Freedman and Zilberstein (2017) is a complementary approach for the interactive Human-aware Intervention model we hope to implement in the next phase of this work. However, we will specially focus on using automated planning to inform the decision making process of the human actors following intervention. In addition, our work in Unsafe Suffix Recognition can be further extended by relaxing the assumptions we have made in the current implementation about the agents and the environment, specifically deterministic actions and full observability for the observer. This may require adopting planning techniques like the one proposed by Amato et al., but with different reward functions. For example, for intervention problems the reward function may take into account the freedom of the actor to reach his goal while ensuring safety.

### 8.3. Dealing With Misconceptions Held by the Actor

In our intervention model the undesirable state is hidden to the user. This is similar to the user having a misconception or a false belief about the domain as the user “believes” the undesirable state is actually safe. Although for our Intervention problem, we assume that the user's belief model is explicitly available to the observer, in other situations this assumption may not hold (e.g., the observer may have limited sensing capabilities). In this case, another agent in the environment (like the observer in our Intervention problem), needs to be able to acquire the beliefs the user has. Talamadupula et al. (2014) discuss a belief acquisition process for a search and rescue domain. The belief acquirer maps the beliefs into a planning problem, allowing him to predict the plan of the agent who is missing the beliefs. The predicted plan and the belief acquirer's own plans are then used to achieve coordination among human-robot teams.

Shvo et al. (2020), in Epistemic Multi-agent Planning, use a multi-agent modal logic to model an observer (and other actors) having different beliefs about the world and other actors. This is in contrast to Talamadupula et al. (2014), who use First-order logic. Given an Epistemic Plan Recognition problem (for an Epistemic Planning observer and an actor), the authors define an *ill-formed* plan with respect to some goal if and only if the plan achieves the goal with respect to the actor but does not achieve the goal from the observer's perspective. The authors highlight a limitation of Epistemic Plan Recognition (also applicable in normal Plan Recognition). The observer's recognition efficacy is dependent on the completeness and the veracity of the observer's beliefs about the environment and the actor. In addition it is also limited by how distinguishable the goals and the plans are that need to be recognized. Our intervention solution attempts to address the problem of improving recognition accuracy when plans are indistinguishable. Shvo et al. (2020) introduce *adequacy* for the recognition process when the actor's actual beliefs are different from the observer's beliefs about the actor. If the observer's beliefs about the actor's beliefs are *adequate*, then the observer can generate precisely all plans that the actor can also generates for some goal that also satisfies the observations.

### 8.4. AI Safety

Using our Intervention models an observer can recognize, with few false alarms/misses, that an undesirable state is developing. The recognition enables the observer to take some action to help the user avoid the undesirable state and complete the task safely. Therefore, our work is also a precursor to incorporating safety into AI systems.

Zhang et al. (2018) use factored Markov Decision Process to model a domain where an agent, while executing plans to achieve the goals that are desirable to a human user, also wants to avoid the negative side effects that the human user would find undesirable/surprising. The agent has complete knowledge about the MDP, but does not know about the domain features that the user has given permission to change. In order to find the safety optimal policies, the agent partitions the domain features as *free, locked* and *unknown* (treated as locked). Then the MDP is solved using linear programming with constraints that prevent the policy from visiting states with changed values for the locked, unknown features. The feature partitioning is similar to our analysis of safe and unsafe plan suffixes using features of plans, where we explore the plan space to recognize what plans enable/satisfy the undesirable state and what do not. In contrast to their model, we model the agents' environment as a deterministic domain using STRIPS. Zhang et al. (2018) policy generation process interacts with the user (through querying) to find the safe-optimal policies that the user really cares about. Saisubramanian et al. (2020) propose a multi-objective approach to mitigating the negative side-effects. Given an task modeled as a MDP, the agent must optimize over the reward for the assigned task (akin to the desirable goal in our Intervention problem), minimize the negative side effects (the undesirable state) within a maximum expected loss of the reward for the assigned task (*slack*) in order to minimize the negative side effect. Being able to handle the negative side effects, caused by imperfect information in the environment is also pertinent to Human-aware Intervention that we propose. Although in this work we are more focused on intervention recognition than intervention response, it's also important to consider how the user's feedback/preferences can be factored into intervention recovery for more robust human-agent interaction.

Hadfield-Menell et al. (2016) introduce *cooperative inverse reinforcement learning* (CIRL) to ensure that the autonomous system poses no risks to the human user and align it's values to that of the human in the environment. The key idea is that the observer (a robot) is interactively attempting to maximize the human's reward while observing the actions executed by the human. The cooperative game environment is modeled as a Partially Observable Markov Decision Process and the reward function incentivizes the human to teach and the robot to learn, leading to a cooperative learning behavior. The problem of finding the optimal policy pair for the robot and the human is found by reducing the problem to solving a partially observable Markov decision process. Intervention is a continuous process where the user and the agent will interact with each other repeatedly until the task is complete Especially in helpful intervention (like the idea we propose in this work, repeated interaction allows the human user and the agent to learn more about the task and hopefully complete it safe-optimally. CIRL formalizes a solution to address this problem.

### 8.5. User Behavior Classification

The design of observers for human users require that the recognizer be able to identify human behavior and how well the behavior aligns with the goals of the system they interact with. Human users are not always rational and may have hidden goals. It may be an unfair comparison to model humans as rational agents in real life scenarios. Behavior classification aims to achieve some insight about the actor from the passive observer's perspective. The work proposed by Borrajo and Veloso (2020) discusses the design of an observer, which tries to learn characteristics other agents (humans and other) by observing their behavior when taking actions in a given environment.

Using the financial transactions domain as a case study, Borrajo and Veloso (2020) models two agents: the actor (e.g., a bank customer) and an observer (e.g., the banking institution). Only the actor can execute plans in the environment. The observer does not know the actor's goal and has partial observability of the actor's behavior (actions the actor executes). Then, the observer's task is to classify the observed behavior into different types of known behavior classes. In order for the application to be domain-independent, the authors use plan distance measures (e.g., Jaccard similarity) between observed actions and distance between observed states as features to train the classifier. We use similar features to recognize the actor's plan prefixes that lead to undesirable states.

### 8.6. Providing Intelligent Help to Human Users Through Intervention

Virvou and Kabassi (2002a,b) discuss the design of a system that provides intelligent help for novice human users while using a file manipulating software application. The Intelligent File Manipulator (IFM) is an online help system where it automatically recognizes that an action may not have the desired goal for the user and offers help by generating alternative actions that would achieve the user's goals. IFM uses a user modeling component to reason over the observed actions. The user modeling component combines a limited goal recognition mechanism and a simulator for users' reasoning based on Human Plausible Reasoning theory to generate hypotheses about possible errors the user might make.

There are some similarities between IFM and our proposed intervention framework. Both models use observations of actions as input for deciding intervention. Both models assume that the user's goals are known. We now discuss some differences between the IFM intervention model and our proposed model: Intervention by Suffix Analysis. The IFM domain is modeled as a task hierarchy, while our domain models (benchmark and Rush Hour) are sequential. To map the user's observed sequence of actions to the plans leading to the desirable and undesirable states, our intervention model uses automated planning to explore the plan space. Then, we analyze the remaining plan suffixes using machine learning to decide intervention. IFM does not use automated planning. Instead it uses a limited goal recognition mechanism called “instability” to identify when users need help. They identify a set of states of the file system as undesirable such as empty directories, multiple copies of a certain file etc. If the file system state contains any of the preset undesirable states, then the system contains instabilities. The user's action will either add an instability or remove an existing one from the system's state. The system tracks the progress of the user's plan(s) by monitoring how the instabilities are added and removed from the system. IFM categorizes the user's observed actions into four categories “expected,” “neutral,” “suspect,” and “erroneous” depending on how compatible the observed actions are with the user's hypothesized intentions. Intervention in IFM takes place when the user executes “suspect” or “erroneous” actions because they signal that there are still unfinished plans. To help the user recover from intervention, the IFM flags “suspect” or “erroneous” actions, and suggests alternative actions that are compatible with the user's intentions. Finding the alternative actions similar to the ones the user has already executed is done based on the user models derived from the Human Plausible Reasoning theory. We hope to address the issue of intervention recovery for our proposed Human-aware Intervention Problem in future developments of our application.

Yadav et al. (2016) present HEALER, a software agent that sequentially select persons for intervention camps from a dynamic, uncertain network of participants such that the spread of HIV/AIDS awareness is maximized. Real-life information about the nodes of the network (human users) are captured and modeled as a POMDP. The Intervention problem discussed in this work is slightly different from our model. Solving the POMDP gives the solution for how to select the most influential individuals from the network to maximize awareness among the population. In contrast, our intervention model is defined for a discrete and sequential environment. A similarity between the models is that they codify properties of actual human users into the POMDP so that the model can be adopted in real-life application. Our Human-aware Intervention model too is designed from actual human user data.

A body of literature on managing task interruption focuses on using cognitive modeling to predict human behavior, which can be used to identify intervention points. Hiatt et al. (2011) apply theory of mind to accommodate variability in human behavior during task completion. They show that a theory of mind approach can help explain possible reasons behind a human's unexpected action, that then allows the robot to respond appropriately. Ratwani et al. (2008) demonstrate that a cognitive model can accurately predict situations where a human missed a step in a sequence of tasks. More recently, Altmann and Trafton (2020) show how to extend a cognitive model to explain a cognitively plausible mechanism for tracking multiple, interacting goals.

### 8.7. Intervention in Cyber-Security for Home Users

Cyber-security domain offers a lot of promise to study behavior both as normal users and as adversaries in automated planning. Behavioral Adversary Modeling System (BAMS) (Boddy et al., 2005) uses automated planning to help computer network administrators in analyzing vulnerabilities in their system against various kinds of attacks. BAMS takes into account the properties of an adversary and produces plans that lead to system exploits that also coincides with the adversary model. While this work does not directly apply to plan recognition at its core, it illustrates a use case where classical planning can be used to design assistive systems targeted toward human end users.

In this work, we take a step toward designing assistive systems to help human end users, who are non-experts (e.g., home users). Home users are specially vulnerable to undesirable consequences because they lack the know-how to recognize risky situations in advance. A previous study (Byrne et al., 2016) showed that home users pay more attention to the benefits of the activities than the risk; they have goals that they want/need to achieve and are willing to take the risk to achieve them. Many triggering actions may be normal activities (e.g., reading email, clicking on links) with the user more focused on the goal than on the risk. Thus, the undesirable consequence recognition problem needs to take into account the user's intention as well as the undesirable consequence.

Howe et al. (2012) observed that most studies that look into computer security practices of users relying on self reported surveys suffered from issues such as respondent bias, socially desirable responding and peer perception. The authors posited that experiments based on simulation, which place the participant in the actual situation that is monitored can help reduce such issues and also be leveraged to assess the emotional reactions of users to interventions and warnings.

The Intervention Problem can be directly applied in the cyber-security domain. An attacker attempting to trick the user into compromising his security/privacy during day-to-day computing tasks (e.g., reading email, installing software) fits the intervention model with the user, competitor and the observer we discussed in this work. Given a cyber-security planning domain model with sufficient complexity (e.g., BAMS domain model), where the undesirable state (i.e., security breach) may develop over time, the Unsafe Suffix Analysis Intervention model can be applied to recognize the threat in advance. A key requirement in helping users in cyber-security domain is to minimize the false positives and negatives during intervention recognition. As evidenced by the experiment results on benchmark domains and the Rush Hour domain confirm, our proposed learning based algorithm addresses this requirement well. While our approach uses Automated Planning, a complementary approach proposed by Roschke et al. (2011) use Attack Graphs to model vulnerabilities in an intrusion detection system to detect attack scenarios while decreasing false positives. However, the intervention recognition must also be paired with intervention recovery in cyber-security domains to ensure the safety of the agent or the human user, particularly when the user has partial visibility or limited capability for understanding the severity of threats. Intervention recovery is also important in help the agent or the human user safely complete the task.

## 9. Discussion

In this paper, we formalized a family of Intervention Problems and showed that how these problems can be solved using a combination of Plan Recognition methods and classification techniques to decide when to intervene. The Unsafe Suffix Intervention Problem uses automated planners to **project the remaining suffixes** and extract features that can differentiate unsafe remaining suffixes from the safe remaining suffixes. In contrast, the Human-aware Intervention Problem uses **only the observed history** *H* to extract features that can separate the solutions leading to undesirable state from the solutions that will avoid it. We compared the Unsafe Suffix Intervention and Human-aware Intervention using the state-of-art Plan Recognition approaches in the literature as the baseline and found that our learning based intervention solutions dominate the existing Plan Recognition algorithms for both benchmark Intervention Problems and a new intervention benchmark, Rush Hour.

In Unsafe Suffix Intervention, when intervention models are trained using features extracted from the Intervention Graph, all the learned models chose distance to u and Risk as the dominant features. We were not able to identify clear dominant features in learned models built with Plan Space Sampling. In Human-aware Intervention, the best performing learned model for *k* = {1, 2, 3}, the logistic regression classifier, selected backtracks, blocks, frees, reset, freebci, freebcd, freegci, freegcd, mgc, mbc and moved as the features for determining intervention.

We show that both the Unsafe Suffix Intervention Problem and the Human-aware Intervention Problem can be re-framed as Plan Recognition problems and use the recognition process to decide intervention. The results prove the feasibility of this approach. Plan recognition approaches are faster at returning a decision in some cases. However, compared to the proposed machine learning based solutions, Plan Recognition based intervention accuracy, precision, and recall are low for both benchmark Intervention Problems and the Rush Hour Intervention Problem. The requirement of setting goal priors for Plan Recognition is an issue that must be overcome for intervention. This is because during execution, the user's plan may be subverted to achieve the undesirable state by environmental factors (such as attackers and hidden knowledge) regardless the priors. In Unsafe Suffix Intervention, we find that even when we assume reasonable goal priors, if the plans for u share long common action sequences with plans for d, Plan Recognition as Planning (PRP) approaches fail to correctly disambiguate between u and d. This affects the overall intervention accuracy by producing many false alarms and misses. In the intervention scenario we simulated with the Rush Hour domain, setting goal priors is even more problematic. This is because the user is informed about the presence of a forbidden vehicle and that the puzzle can be solved without moving it prior to executing the planning task. The result of that information being given to the user is that the goal prior for u is low compared to d. In our experiments, we show that the recognition accuracy for PRP approach drops when goal priors are set this way. We remove the dependency on goal priors by using features of projected remaining suffixes (in Unsafe Suffix Recognition) and the observed partial solution *H* (in Human-aware Intervention) to learn the differences between safe and unsafe plans from example training data and using the learned models to predict intervention.

We observed that different features (or their combinations) affect the intervention decision in the benchmark domains. Next, we describe which features were selected by the intervention recognition classifiers for the Intervention Graph and the Plan Space Sampling methods.

- **Blocks-1:** When Intervention Graph features are used, the decision tree, k-nearest neighbor and naive Bayes classifiers select distance to u to make the intervention decision. The logistic regression classifier selects distance to u and Risk to make the intervention decision. When using the Plan Space Sampling method, the decision tree, logistic regression, naive Bayes classifiers select the Landmark Completion Heuristic. The k-nearest neighbor classifier selects the median Causal Link Distance between the reference plan and the plans to u.
- **Blocks-2:** When Intervention Graph features are used, the decision tree classifier selects Risk to decide intervention. The decision tree, logistics regression and naive Bayes classifier use Risk, Desirability, distance to u and d, and the active attack landmark percentage collectively to decide intervention. When deciding intervention using the Plan Space Sampling method, the decision tree uses the median action set distance to u, minimum generalized edit distance for state sequences for u and median causal link distance to d features. The k-nearest neighbor, logistic regression and naive Bayes classifiers use all features in the Sampled Feature Vector.
- **EasyIPC:** When Intervention Graph features are used, the decision tree, k-nearest neighbor and naive Bayes classifiers select distance to u to make the intervention decision. The logistic regression classifier selects Risk to make the intervention decision. When deciding intervention using the Plan Space Sampling method, the decision tree and the naive Bayes classifiers use the Landmark Completion Heuristic. The k-nearest neighbor classifier selects the median Causal Link Distance between the reference plan and the plans to u. The logistic regression classifier uses several features: action set distance, causal link distance, state sequence distance, minimum remaining distance, minimum edit distance to u, and state sequence distance, minimum remaining distance to and minimum edit distance of action and state sequences for plans leading to d.
- **Ferry:** When using Intervention Graph features, all four classifiers select Risk to make the intervention decision. When deciding intervention with the Plan Space Sampling method, the decision tree classifier selects median causal link distance to u, median state sequence distance to u, minimum remaining distance to d and minimum edit distance to d as features. The k-nearest neighbor classifier selects the median state sequence distance and the minimum remaining distances to u to make the intervention decision. The logistic regression classifier uses the action set distance, causal link distance and state sequence distance, minimum edit distance in action and state sequences to u. In addition the classifier also uses the state sequence distance and minimum edit distance to d. The naive Bayes classifier uses action set distance and causal link distance to d, in addition to causal link distance to u, state sequence distance to u, edit distances for action and state sequences for u, and the Landmark Completion Heuristic.
- **Navigator:** When Intervention Graph features are used all four classifiers select Risk to make the intervention decision. When deciding intervention with the Plan Space Sampling method, the decision tree, k-nearest neighbor and naive Bayes classifiers use the Landmark Completion Heuristic. The logistic regression classifier use the action set distance, causal link distance, state sequence distance, minimum remaining distance to critical, minimum edit distance for state sequences and the Landmark Completion Heuristic.

We model intervention by recognizing the directly contributing actions using the Blocks-1, EasyIPC, Ferry and Navigator domains. For the Blocks-1 problems, when using the Intervention Graph feature vector, the observer can recognize when intervention is required by monitoring one or two features (e.g., Risk and remaining distance to u). A similar observation can be made when using the Plan Space Sampling feature vector (the Landmark Completion Heuristic, Causal Link Distance). The EasyIPC intervention problems also use a few features (e.g., the remaining distance to u and Risk) for deciding whether to intervene. The logistic regression classifier uses more features from the Plan Space Sampling feature vector compared to the other three classifiers to decide intervention for the EasyIPC domain. The intervention decision for the Ferry domain and the Navigator domain rely only on the Risk feature when using the Intervention Graph feature vector. For the Ferry domain, intervention using the Plan Space Sampling feature vector require monitoring for many features. In contrast, for the Navigator domain three classifiers rely on the Landmark Completion Heuristic to decide intervention. The logistic regression classifier use many plan distance metrics in addition to the Landmark Completion Heuristic to make the intervention decision.

We use the Blocks-2 domain to model intervention by recognizing indirectly contributing sequences. For this case, more features from the Intervention Graph feature vector is required to make the intervention decision. The same can be observed when using the Plan Space Sampling feature vector.

Our findings comparing PRP and the learned model for Human-aware Intervention show that deciding to intervene based on plan cost differences (PRP) is not sufficient, especially when intervening human users. As seen from the solution length distributions in Section 11.3, the longer the human user spends exploring the state space, the higher the likelihood that his partial plan will get closer to u. Therefore, we argue that intervention for human users require representations that capture characteristics of the actor's behavior in addition to the planning representations. The features based on actions and the features based on state extracted from *H* we propose for learning Human-aware Intervention capture the behavior patterns of the human user and as a result produce accurate learned models. However, a limitation of our proposed approach is that some features in the feature vector are domain-specific. Therefore, adopting the proposed approach in different planning domains may require feature engineering.

There are other methods one could use for generating $X_{◇}$, which we will explore in future work. In Unsafe Suffix Intervention, we generated $X_{◇}$ from the *s*_*H*_ using an automated planner without considering the observations in *H*. We then compared the suffixes in $X_{◇}$ to an observation compatible reference plan. Ramırez and Geffner (2009), Ramırez and Geffner (2010), and Sohrabi et al. (2016a) propose methods to compile the observations into the domain theory, which allows the planner to find observation compatible plans. We can use this technique to also find observation compatible suffixes in $X_{◇}$. By making both the $X_{◇}$ and the reference plan compatible with the observations, we believe the plan distance features will be more accurate for the Plan Space Sampling method.

## 10. Closing Remarks

Intervention is a necessity for online assistive agents and safety critical decision making, where an observer determines how to guide a user toward a desirable outcome while avoiding undesirable outcomes. We propose Intervention as a solution to this problem and introduced two algorithms that combine automated planning and machine learning to decide whether or not the user's likely plan will avoid the undesirable state. Representing the user's task as a planning problem allows us to extract features of the user's plan space that can be used to produce learned models to recognize when intervention is required. Our first solution Unsafe Suffix Intervention, uses automated planning to project the remaining suffixes and extract features to differentiate between safe suffixes that avoid the undesirable outcome and unsafe suffixes that do not avoid the undesirable outcome. The second solution, Human-aware Intervention uses only the observed plan to extract features that can differentiate between safe and unsafe solutions. We showed that the two learning based intervention solutions dominate the state-of-the-art Plan Recognition algorithms in identifying when intervention is required.

In this work, our objective was to identify, given a sequence of observations, whether the user requires intervention while minimizing false positives and negatives. For our current implementation we assumed that the intervention comes in the form of a block or an alert message. The natural next step following intervention is helping the user decide what to do next. This extension is particularly important for observers where the user is a human user who would like to be guided toward the goal instead of being given the solution outright (e.g., an automated tutoring agent). We identify two sub-problems in helping the user decide what to do next. First, we can explore how automated planning can be used to gradually probe the search space of the remaining planning task following intervention. In certain cases, the user may want a quick, well-focused help. For example, in the Rush Hour puzzle, the observer can suggest the first move in the shortest remaining plan that avoids the forbidden vehicle as a hint after intervening. In other cases, the user would prefer more abstract suggestion. For example, in the Rush Hour puzzle, the observer can suggest the vehicles that must be moved to solve the puzzle (i.e., the landmarks of the planning task). In various stages of the puzzle solving task, the user may opt to use these suggestions differently. The second sub problem is explaining intervention and the follow-up to intervention. We can explore how effective different explanation models (e.g., contrastive, selective) are in explaining intervention and intervention Recovery to human users.

There are several other extensions to our current intervention framework, that we would like to explore as future work. In the planning domains we used to model intervention tasks, we assumed that the user's actions are deterministic and there is only one undesirable state that needs to be avoided. It is possible to relax these two assumptions and explore intervention in non-deterministic environments where the user needs to avoid multiple undesirable goals. However, in order for intervention to be meaningful, the intervention planning domains need to be descriptive enough to model complex tasks. We can explore the feasibility of adopting scenario building game environments like Minecraft for this purpose.

An interesting issue from our Rush Hour intervention study is how well the classifiers trained from user data would generalize to larger and more difficult Rush Hour puzzles. For example, the puzzle can be made difficult by adding more forbidden vehicles, more vehicles, and also introducing random exit points in the board. Addressing this question is left for future work.

## Data Availability Statement

The datasets presented in this study can be found in online repositories. The names of the repository/repositories and accession number(s) can be found below: https://github.com/sachinisw/intervention_datasets/.

## Ethics Statement

The studies involving human participants were reviewed and approved by Institutional Review Board (IRB), Colorado State University. The participants provided their informed consent online to participate in this study.

## Author Contributions

SW is a Ph.D. student in the Computer Science Department at the Colorado State University conducting research on automated planning. SW's contributions to this work are, concept development, coding, experimental design, data analysis, and writing. DW is a Professor of Computer Science at Colorado State University. His research focuses on search, evolutionary computation and machine learning. He is a Fellow of the ACM. DW's contributions to this work are concept development and writing. MR is a Research Scientist at NRL's Navy Center for Applied Research in AI (NCARAI, Code 5514). His research experience spans a cross section of applied planning, scheduling, and machine learning for decision support. MR's contribution to this work is writing. All authors contributed to the article and approved the submitted version.

## Funding

The research was funded by AFOSR, NRL, and ONR. SW was also funded by a graduate assistantship from Colorado State University.

## Conflict of Interest

The authors declare that the research was conducted in the absence of any commercial or financial relationships that could be construed as a potential conflict of interest.

## Publisher's Note

All claims expressed in this article are solely those of the authors and do not necessarily represent those of their affiliated organizations, or those of the publisher, the editors and the reviewers. Any product that may be evaluated in this article, or claim that may be made by its manufacturer, is not guaranteed or endorsed by the publisher.

## Footnotes

^1^ http://www.cs.waikato.ac.nz/ml/weka/

## 11. Appendix

We discuss details about the Rush Hour human subject study and the findings. The following sections present the evidence on which we based the design choices for the Rush Hour planning task. The sections are cross-referenced throughout the main content.

### 11.1. Rush Hour Human Subject Study: Pilot Experiment

Before the study was actually administered to our recruited subjects, a pilot study was conducted with nine graduate students to assess whether they would be able to solve the puzzles within a reasonable amount of time. The pilot study participants solved their assigned puzzles within 5–10 min. The pilot study participants were also interviewed informally to get their perception of the puzzle difficulty. The participants commented that the puzzles were “*challenging*” and “*forced me to think*”. The same puzzles used in the pilot were used in the actual study.

### 11.2. Demography Survey Findings

39 participants were below the age of 20, while 38 subjects were between the age 20-25. Maximum age was 41 years. 70 of the 117 participants were male. When asked if they liked puzzle solving tasks, 78% of the participants either agreed or strongly agreed with the statement. Specifically to the Rush Hour game 79% of the participants liked or strongly liked Rush Hour. The most common reason as to why the participants liked puzzle solving tasks was that puzzle solving stimulates critical thinking skills. 30% of the participants usually did a puzzle solving task once a month, while 21% of the participants solved a puzzle once a week. When asked about strategies the participants used to solve difficult puzzles, 79% of the group said that they kept trying until the puzzle was eventually solved, while 12% of the participants said that they would ask for help.

Given a new puzzle that they have not seen before, if they get stuck while solving the puzzle, 26% said that they would not like any outside help. 47% of the participants said that they would like a suggestion/tip that would get them past the current situation. 15% said that they would like a warning, which indicated that their current approach would lead to a dead-end. 8% of the participants said that they would like a warning and an explanation to help them prevent getting stuck in the future. The most common medium for solving puzzles was using their mobile devices (42%). 31% of the participants used the personal computers/laptops to solve puzzles. 19% of the participants solved puzzles using physical means (e.g., puzzle books, newspapers and physical puzzles such as Rubik's cubes).

### 11.3. User Solutions Grouped by Length

For each puzzle, we sorted the solutions by the number of moves (in the complete solution) in the ascending order and split them into three groups (fast, medium, slow). We ensured that the three groups for each puzzle contained approximately equal number of users. Table A1 summarizes the findings. There were 46 users in the fast group, 42 in the medium and 48 in the slow group. Mean refers to the mean number of moves in a solution produced by users who solved a specific puzzle. Forbidden moves refers to the number of times, the users who solved a specific puzzle moved the forbidden vehicle.

**Table A1:** Plans produced by human users grouped by the mean number of moves and the number of forbidden moves.

**PID**	**Fast (46)**		**Medium (42)**		**Slow (48)**	
	**Mean**	**Forbidden** ** moves**	**Mean**	**Forbidden** ** moves**	**Mean**	**Forbidden** ** moves**
P1	25.5	0	41.7	0	64.7	0
P2	74.7	4	137.7	16	277	28
P3	26.5	8	36.2	9	43.7	6
P4	25.2	1	32	4	76	28
P5	18.3	3	26	2	44.75	13
P6	22	0	24.5	0	39.6	0
P7	38.5	5	53.3	16	120	37
P8	9	0	9	0	10	0
P9	27.8	8	48.6	12	99	28
P10	50.3	11	66	14	127.3	46
